# Characterizing the quick-killing mechanism of action of azithromycin analogs against malaria parasites

**DOI:** 10.1128/aac.01783-24

**Published:** 2025-07-25

**Authors:** Emma Y. Mao, William Nguyen, Gouranga P. Jana, Bikash C. Maity, Samuel Pazicky, Carlo Giannangelo, Janette Reader, Mufuliat T. Famodimu, Lyn-Marie Birkholtz, Michael J. Delves, Darren J. Creek, Zbynek Bozdech, Benoît Laleu, Jeremy N. Burrows, Brad E. Sleebs, Maria R. Gancheva, Danny W. Wilson

**Affiliations:** 1 Research Centre for Infectious Diseases, School of Biological Sciences, The University of Adelaide 110455, Adelaide, Australia; 2 ARC Training Centre for Environmental and Agricultural Solutions to Antimicrobial Resistance (CEA StAR), Brisbane, Australia; 3 Institute for Photonics and Advanced Sensing (IPAS), The University of Adelaide 1066, Adelaide, South Australia, Australia; 4 Walter and Eliza Hall Institute of Medical Research 5388, Melbourne, Victoria, Australia; 5 Department of Medical Biology, The University of Melbourne 2281, Melbourne, Victoria, Australia; 6 TCG Lifesciences Private Limited, Salt-lake Electronics Complex 30151 , Kolkata, India; 7 School of Biological Sciences, Nanyang Technological University 219572, Singapore, Singapore; 8 Monash Institute of Pharmaceutical Sciences, Monash University 2541, Melbourne, Victoria, Australia; 9 Department of Biochemistry, Genetics and Microbiology, University of Pretoria 598960, Pretoria, South Africa; 10 London School of Hygiene and Tropical Medicine 4906, London, United Kingdom; 11 MMV Medicines for Malaria Venture, ICC, Geneva, Switzerland; 12 Burnet Institute 104125, Melbourne, Victoria, Australia; The Children's Hospital of Philadelphia, Philadelphia, Pennsylvania, USA

**Keywords:** malaria, antimalarial agents, *Plasmodium*, azithromycin, cellular thermal shift assay

## Abstract

Drug resistance is steadily undermining the efficacy of frontline anti-malarials, highlighting the urgent need for novel therapies with alternative mechanisms of action. The chemical addition of different moieties to azithromycin yields compounds with improved quick-killing potency against malaria parasites, with the most active analogs typically containing a chloroquinoline group. Here, we investigated the quick-killing mechanism of five azithromycin analogs, two of which contain differentially oriented chloroquinoline moieties. The improvement in quick-killing activity over azithromycin for non-chloroquinoline analogs was around 10 -to 42-fold, with chloroquinoline-containing analogs showing a further 2- to 17-fold improvement over non-chloroquinoline compounds. Chemical inhibition of hemoglobin digestion and chloroquine’s inhibitory effect against heme polymerization linked analogs with both chloroquinoline and non-chloroquinoline modifications to a chloroquine-like mechanism of action. However, none of the analogs showed a significant reduction in efficacy against chloroquine-resistant asexual blood-stage parasites. Multiple attempts at selecting for azithromycin analog-resistant parasites to elucidate the mechanism of quick-killing were unsuccessful. Application of cellular thermal shift proteomics revealed that azithromycin analogs significantly stabilized 34–155 different proteins in trophozoites, a high number that showed minimal overlap with chloroquine. Additionally, our most potent chloroquinoline-containing analog demonstrated a significant improvement in gametocytocidal activity over azithromycin and further maintained moderate inhibition of chloroquine-insensitive late-stage gametocytes. These findings support that this class of azithromycin analogs kills malaria parasites through a broad range of potential mechanisms, making them promising candidates for optimization as fast and broad-acting anti-malarials.

## INTRODUCTION

Malaria remains a major global health challenge, imposing a significant economic burden on many affected countries. In 2023 alone, malaria parasites caused an estimated 263 million new infections and 597,000 deaths, the majority of which were attributable to *Plasmodium falciparum* infections in children under 5 years of age in Sub-Saharan Africa (1). While the widespread implementation of control measures, such as insecticide-treated nets, indoor residual spraying, and anti-malarials, contributed to significant reductions in global mortality rates, these figures have alarmingly stalled in the last decade, in part due to the spread of drug-resistant parasites (2, 3). Of great concern is resistance to our current frontline anti-malarial treatments, artemisinin combination therapies (ACTs), which has now emerged in several malaria-endemic regions and threatens increased treatment failure (4, 5). Therefore, new anti-malarial agents with alternative mechanisms of action and no cross-resistance to existing therapies are urgently needed.

Since the discovery of the malaria parasite’s apicoplast, a plastid organelle with bacterium-like ribosomal complexes (6), antibiotics have been explored for repurposing as potential anti-malarial agents (7). One such drug is azithromycin, which is a clinically used macrolide antibiotic. Due to its long half-life of ~68 hrs (8) and considerable safety and tolerability in children and pregnant women (9–11), azithromycin has been investigated as a prophylactic and partner drug in malaria combination therapies (12–15). However, there has been limited progress into clinical implementation as azithromycin’s efficacy in its current form remains inferior when compared to existing clinically used drugs, such as doxycycline (15, 16) and artesunate plus mefloquine (17).

Azithromycin works against malaria parasites by inhibiting protein synthesis in the bacterium-like ribosomes of the apicoplast (18). Parasites treated with azithromycin exhibit a “delayed-death” phenotype (120 hr IC_50_ = ~0.05 µM, chloroquine-sensitive 3D7 line), whereby parasite killing occurs during the second replication cycle due to inheritance of a defective apicoplast incapable of producing essential metabolites required for survival (7). At higher treatment concentrations (48 hr IC_50_ = ~8 µM, chloroquine-sensitive 3D7 line), azithromycin has demonstrated the ability to inhibit merozoite invasion of red blood cells (RBCs) and rapidly induce parasite death within the first replication cycle through an apicoplast-independent mechanism, termed “quick-killing” (7, 19). The potency of this quick-killing can be significantly improved through the addition of different functional groups to azithromycin (20–24), with some modifications that confer improved quick-killing activity also maintaining the drug’s delayed-death activity. This opens the possibility of developing a dual-acting anti-malarial with both therapeutic and prophylactic potential. Other modifications, namely, those on the desosaminyl carbohydrate of azithromycin, can abrogate delayed-death activity by blocking ribosome binding within the peptide exit tunnel (18, 25–27). These single-acting azithromycin analogs may be preferential in some cases for human treatment as they minimize the risk of off-target antibiotic activity.

Previous work with analogs of azithromycin has found that the addition of chloroquinoline functional groups to the azalide scaffold tends to result in compounds with the greatest quick-killing potency (22, 24, 28). Early studies have pointed toward a quick-killing mechanism resembling chloroquine’s inhibition of hemoglobin digestion and heme polymerization in the parasite’s digestive vacuole (24, 28, 29). However, there are also conflicting data suggesting several dissimilar properties to chloroquine, these being the following: (i) the lack of activity on heme and hemozoin levels in whole cell-based assays (22); (ii) minimal evidence of a chloroquine-like metabolomics signature upon treatment (22); (iii) ability to inhibit merozoite invasion of RBCs where there is no hemozoin crystal and chloroquine is not active (19, 22); (iv) potent and rapid early ring-stage killing activity where chloroquine is typically not very active (21, 22); (v) minimal reduction in drug potency against chloroquine-resistant parasites (20, 22, 29).

**Fig 1:**
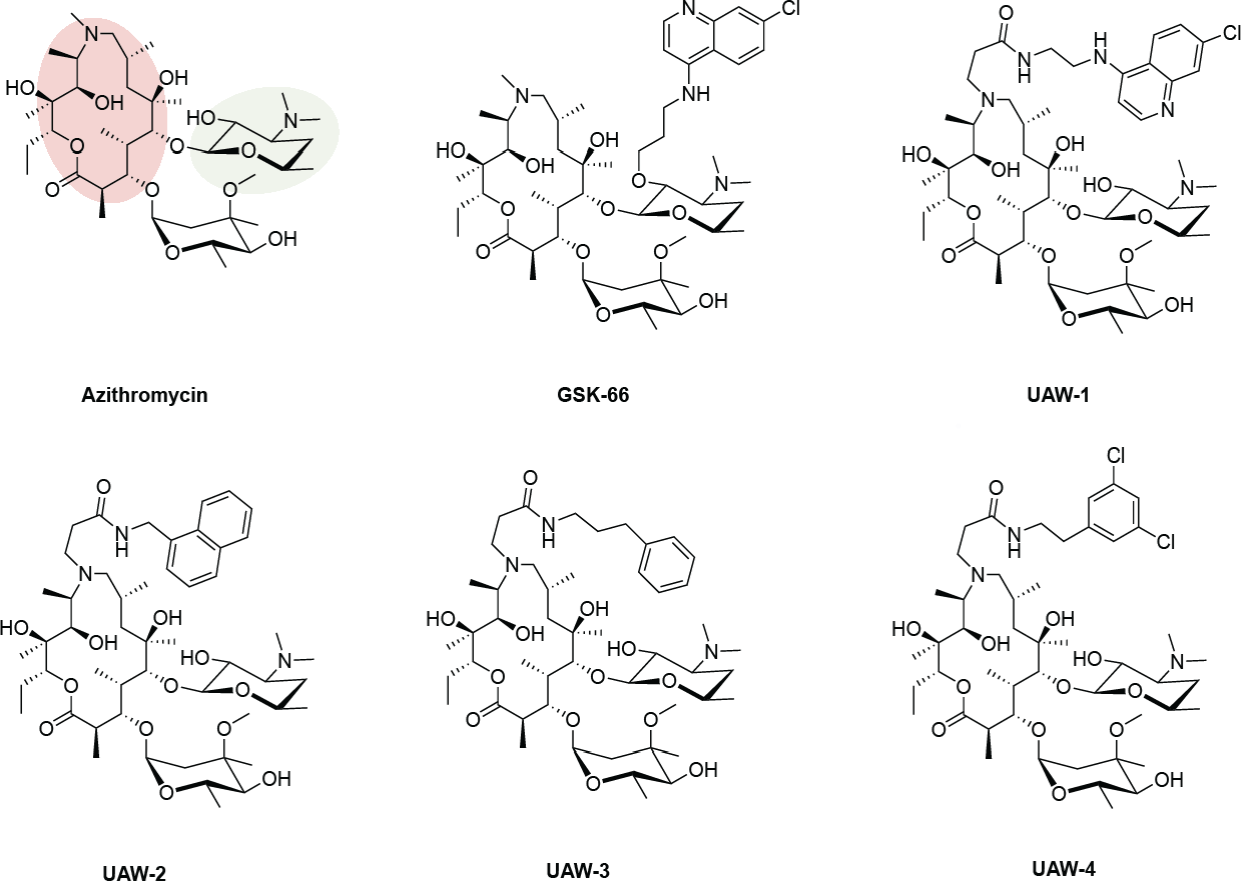
Chemical structures of azithromycin and analogs used in this study. Compounds used in this study possess chemical modifications to azithromycin at either the O-position of the desosaminyl carbohydrate in green (GSK-66) or the N6-position of the macrolactone ring in red via an amide linkage (UAW-1-4). GSK-66 and UAW-1 have added chloroquinoline moieties, whereas UAW-2,-3, and −4 have non-chloroquinoline functional groups. UAW-2 contains an added naphthalene group, UAW-3 a phenyl group, and UAW-4 a 3,5-dichlorophenyl group.

In this study, we characterized the quick-killing activity of five azithromycin analogs and investigated the underlying mechanisms driving their enhanced quick-killing potency over azithromycin. One of these analogs has previously been explored (GSK-66 [22, 24]), whereas the remaining four (UAW-1,-2, −3, and −4) are novel with no published data about their quick-killing or delayed-death activity. All analogs exhibited improved quick-killing activity over azithromycin against a range of chloroquine-sensitive and -resistant *P. falciparum* parasites. Using synergy and β-hematin formation assays, we explored the contribution of a chloroquine-like inhibition of hemoglobin digestion and heme polymerization to the quick-killing activity of analogs with chloroquinoline (GSK-66 and UAW-1) and non-chloroquinoline modifications (UAW-2,-3, and −4) (Fig. 1). Our findings show that both chloroquinoline and non-chloroquinoline-modified azithromycin analogs exhibit chloroquine-like anti-malarial properties. However, the data also support that the mechanism of quick-killing encompasses a broader range of potential targets, allowing for the maintenance of quick-killing potency in multiple chloroquine-resistant *Plasmodium* lines, as well as additional inhibitory activity against the chloroquine-insensitive late-stage gametocytes for GSK-66.

## MATERIALS AND METHODS

### Compounds

GSK-66 was synthesized by TCG Lifesciences (synthesis originally described in (24) as compound 22), while the UAW azithromycin analogs were synthesized by the Walter and Eliza Hall Institute of Medical Research, as described in File S1. Stock concentrations of all azithromycin analogs (10 mM, GSK-66 & UAW compounds), E64 (50 mM, Sigma-Aldrich), deferoxamine mesylate (DFO) (100 mM, Sigma-Aldrich), and verapamil hydrochloride (1 M, Sigma-Aldrich) were solubilized in dimethyl sulfoxide (DMSO), whereas chloroquine diphosphate salt (10 mM, Sigma-Aldrich) was dissolved in 10% acetic acid in H_2_O. Azithromycin (10 mM, AK-Scientific) was solubilized in DMSO for the majority of its applications, except for Pf3D7 isobolograms, where it was instead made up to 25 mM in 100% ethanol to account for a higher IC_50_ value in this parasite line, which would otherwise be affected by DMSO toxicity at the required working concentrations. The potential variability in azithromycin activity with different solvents was evaluated by comparison of IC_50_ values, which showed no significant difference in potency in our assays (DMSO IC_50_ = 4.6 µM; ethanol IC_50_ = 3.2 µM). All drugs were added to growth assays such that the vehicle was diluted >800 fold for DMSO and >200 fold for 100% ethanol to minimize the risk of non-specific inhibition. A vehicle control was included in all assays to quantitatively assess toxicity.

### Culture and synchronization of asexual blood-stage *Plasmodium* species parasites

*P. falciparum* 3D7, Dd2 (wild-type and hypermutating line), W2mef, 7G8, and Cam3.1^ART resistant (R539T)^ parasites were cultured in human O^+^ type red blood cells (Lifeblood, Australia) at ~3% hematocrit in RPMI-HEPES culture media (pH 7.4, 50 µg/mL hypoxanthine, 25 mM NaHCO_3_, 20 µg/mL gentamicin, and 0.5% Albumax II [Thermo Fisher Scientific]). Cultures were maintained at 37°C in sealed boxes with a gas composition of 1% O_2_, 5% CO,_2_ and 94% N_2_, according to established protocols (30). For use in assays, parasites were synchronized to ring stages by a 10 min treatment with 5% wt/vol D-sorbitol (Sigma-Aldrich).

### Drug inhibition assays and SYBR DNA staining

Sorbitol-synchronized ring stage parasites were prepared to 1% hematocrit in culture media at 1% parasitemia for drug inhibition assays. An 8-point 1:2 dilution series of compounds was added to 90 µL of parasites in a 96-well U-bottom plate to a final volume of 100 µL. Parasite growth from drug inhibition and synergy assays was quantified by SYBR DNA staining (31, 32). Briefly, following the 72 hr incubation period, when parasites were at the late trophozoite/schizont stages in the next replication cycle, the supernatant was removed from each well and the pellet was resuspended in PBS. An equal volume of SYBR Safe DNA Gel Stain Reagent (Invitrogen) in SYBR lysis buffer (pH 7.5, 20 mM TRIS, 5 mM EDTA, 0.008% saponin (wt/vol) 0.08% Triton X100 (v/v)) (1:5,000) was added to each sample well. After a 45 min incubation in the dark, plates were read on a fluorometer (BMG LabTech PHERAstar FS, excitation, 485 nm; emission, 520 nm). Background fluorescence of uninfected RBCs was subtracted, and all wells were normalized to parasite growth in the absence of the drug to calculate the percentage growth of drug-treated parasites. Drug IC_50_s were determined on GraphPad Prism (version 10.0.2) by nonlinear regression of a log(inhibitor) vs response (three parameters) curve, and the statistical significance was calculated using a parametric unpaired T test with Welch’s correction. All IC_50_ values were determined from three or more biological replicates, each conducted with technical duplicates.

### Drug synergy assays and isobologram analysis

Synergy assays were performed using a previously described modified fixed-ratio method (33). Here, synchronized ring-stage *P. falciparum* 3D7 and Dd2 parasites were prepared at 2% hematocrit and 1% parasitemia and treated with a 7-point 1:2 dilution series of two drugs mixed at different ratios (drug A: drug B; 5:0, 4:1, 3:2, 2:3, 1:4, and 0:5), each starting at 8 x IC_50_ to allow their individual IC_50_s to fall at approximately the fourth serial dilution. Parasites (50 µL) were incubated with 50 µL of drug diluted in media for 72 hrs in a 96-well U-bottom plate, and parasite growth was measured by SYBR DNA staining, as described above. Drug interactions with E64 and DFO were performed in chloroquine-sensitive 3D7 parasites, whereas interactions with verapamil were performed in chloroquine-resistant Dd2 parasites.

IC_50_s were determined for each drug individually at their respective proportions added to the drug mix (drug A 100%, 80%, 60%, 40%, and 20%, and vice versa for drug B). All IC_50_ values were determined from three or more biological replicates, each conducted with a single technical replicate. These values were used to calculate the fractional inhibitory concentration (FIC_50_) for each drug combination using the equation below (and vice versa for drug B):


$$FIC_{50}\;\left(drug\;A\right)=\frac{IC_{50}\;\left(drug\;A\;in\;the\;presence\;of\;x\;conc.\;of\;drug\;B\right)}{IC_{50}\;\left(drug\;A\;alone\right)}$$

The FIC_50_ values determined for each ratio of drug combination were averaged across three or more independent experiments and plotted as an isobologram for drug A vs drug B, where concave curves below the dotted additivity reference line generally indicate synergy and convex curves above the additivity reference indicate antagonism. The mean sum of the FIC_50_s (where ${\Sigma FIC}_{50}s={FIC}_{50}\left(drugA\right)+{FIC}_{50}\left(drugB\right)$) was further calculated to provide a quantitative representation of these interactions. As previously implemented to account for inherent experimental variation (34–36), drug combinations exhibiting a mean ΣFIC_50_ <0.8 were considered synergistic, those exhibiting a mean FIC_50_ ≥1.4 were antagonistic, and if equal to 0.8–1.4 were additive.

### Selection of drug resistance in a *P. falciparum* Dd2 hypermutating line

Drug resistance selection was set up in six-well plates with an asynchronous parasite population at 5% parasitemia and 3% hematocrit in 4 mL culture media per well like previously published methods (37). GSK-66 was added to a hypermutating clone of Dd2 parasites harboring mutations in the DNA polymerase δ (38), Dd2-Polδ-D5, at either 10 x or 2 x IC_50_ (IC_50_ in Dd2 parasites = 0.029 µM, Table 1). An equal concentration of compound vehicle was added to another well of Dd2 hypermutating parasites to account for unrelated mutations. Cultures were maintained on drug, with the media and drug replaced every second day, until Giemsa-stained thin blood films revealed that parasites began to die. Following drug removal, parasites were fed every 1–2 days, with 25% of culture replaced with fresh RBCs once a week to allow recovery. GSK-66 was added again once the parasite population recovered, and this drug-cycling was repeated 2–4 times before examination of IC_50_ change in drug inhibition assays. If no recrudescent parasites were observed for more than 1 month after drug removal, the culture was discarded. Parasites surviving cyclic treatment with 2 x IC_50_ of GSK-66 were further subjected to another cycle of treatment at 3 x IC_50_ in an attempt to enhance resistance to GSK-66.

**TABLE 1:** Mean IC_50_ values of chloroquine (CQ), azithromycin (AZR), and azithromycin analogs in 72 hr SYBR Green growth assays against chloroquine-sensitive asexual blood-stage *P. falciparum* 3D7 and a panel of chloroquine/multidrug-resistant *P. falciparum* parasites^*a*^

Compound	IC_50_ (± SEM) μM				
	Pf 3D7	Pf multidrug-resistant lines			
		W2mef	Dd2	7G8	Cam3.I ^*b*^
CQ	0.04 (0.01)	0.23 (*0.07*) ^**^	0.13 (*0.03*) ^*^	0.24 (*0.03*) ^*^	0.18 (*0.05*) ^*^
AZR	4.60 (0.58)	0.26 (0.08) ^*^	0.58 (0.09) ^*^	0.73 (0.097) ^*^	0.39 (0.098) ^*^
GSK-66	0.03 (0.004)	0.03 (*0.01*) *^c^*	0.03 (*0.01*) *^c^*	0.07 (*0.03*) *^c^*	0.06 (*0.02*) *^c^*
UAW-1	0.05 (0.004)	0.07 (*0.002*) *^c^*	0.06 (*0.01*) *^c^*	0.11 (*0.02*) *^c^*	0.06 (*0.01*) *^c^*
UAW-2	0.11 (0.01)	0.04 (*0.01*) ^**^	0.04 (0.007) ^*^	0.07 (*0.01*) *^c^*	0.09 (*0.02*) *^c^*
UAW-3	0.48 (0.06)	0.08 (*0.03*) ^**^	0.16 (*0.04*) ^**^	0.15 (*0.04*) ^**^	0.19 (0.02) ^*^
UAW-4	0.14 (0.01)	0.10 (0.06) ^*c*^	0.08 (0.01) ^*^	0.14 (*0.02*) *^c^*	0.05 (0.02) ^*^

^*a*^ Data represent the mean ± SEM, *n* ≥ 3.

^*b*^ Artemisinin-resistant Cam3.I (R539T) parasites.

^*c*^ No significant IC_50_ difference relative to 3D7, where *P> 0.05*. ^*^ Significant change in IC_50_ relative to 3D7, where *P* < 0.05; ^**^ *P* < 0.009.

### β-hematin polymerization assays

The ability of our test compounds to inhibit β-hematin polymerization was assessed in an *in vitro* propionate buffer system, according to previously published methods (39). All compounds were tested at 1 µM in two independent experiments. Briefly, the test compounds (1 mM) were diluted in 1 M propionate buffer (pH 5.2) containing 20% DMSO, and then 180 µL of each compound was added to deep 96-well plates (Waters) and serially diluted in 90 µL propionate buffer. Freshly prepared hemin solution (5 µL at 2 mM in 0.1 M NaOH) was added to all wells, followed by 5 µL of the lipid catalyst phosphatidylcholine (0.5 mg/mL in propionate buffer) to initiate β-hematin polymerization. Each well was mixed thoroughly by pipetting. Wells containing propionate buffer, hemin, and lipid catalyst alone acted as the positive control for 100% β-hematin formation. Wells that contained no lipid catalyst acted as the negative control for no β-hematin formation. The test plate was incubated for 16 hrs at 37°C with gentle agitation to allow β-hematin crystal polymerization. After 16 hrs, crystal growth was terminated with the addition of 50 µL of 7.5% SDS, followed by thorough mixing by pipetting. The plate was incubated at room temperature for 10 mins with gentle agitation and then centrifuged at 400 × *g* for 3 mins to pellet any crystals formed. The supernatant (50 µL) from each well was transferred to a new 96-well flat bottom plate containing 200 µL of 2.5% SDS and the absorbance detected at 405 nm on an Ensight Plate Reader (Perkin Elmer). The mean absorbance value of propionate buffer alone was subtracted from each reading, and the values were normalized to the positive and negative controls. Percent inhibition of β-hematin polymerization was plotted against the log inhibitor concentration and the data analyzed by nonlinear regression [log(dose) vs response] in GraphPad Prism (version 9.3.1) to yield the concentration that inhibited crystal growth by 50% (IC_50_). All IC_50_ values were determined from two or more biological replicates, each conducted with technical duplicates.

### Mass spectrometry-coupled cellular thermal shift assay (MS-CETSA) sample preparation

Sample preparation was performed as previously described (40). In short, for lysate CETSA, the synchronized *P. falciparum* 3D7 strain trophozoites were released with 0.01% saponin (6 mins, 4°C), washed 2 x with PBS, and resuspended in lysis buffer (100 mM HEPES pH 7.5, 0.2 mM activated Na_3_VO_4_, 20 mM MgCl_2_, 10 mM beta-glycerophosphate, and an EDTA-free protease inhibitor cocktail). The isolated parasites were flash-frozen and thawed 3 x and cryolyzed (Precellys Evolution Touch, 6000 RPM, 6 × 5 sec cycle with 25 sec pause, 4°C) and incubated upon addition of 1% 10:1 n-dodecyl-β-D-maltoside/cholesterol hemisuccinate (DDM/CHS ). The insoluble fraction was separated by centrifugation (20,000 × *g*, 20 mins), the soluble fraction was recovered, and the protein concentration adjusted to 2 mg/mL. The sample was split to 10 tubes of 100 µL and incubated with a concentration series of the drug (9 tubes of 4 x dilution series starting at 100 µM and 1 x DMSO control) for 1 h. The samples were then split into four 25 µL series of tubes; heated to 37, 53, 59, or 65°C for 3 mins; and cooled down to 4°C immediately.

For intact-cell MS-CETSA, synchronized trophozoites were enriched (>90%) by magnetic sorting, the cell density was adjusted to 10 × 10^6^ cells/mL, and after 1 hr of recovery, the culture was split to 10 wells of 4 mL volume and incubated for 1 h with a concentration series of the drug (9 samples of 4 x dilution series starting at 10 µM and 1 DMSO control). Then, the individual samples were centrifuged (1,200 × *g*, 5 mins), resuspended in 100 µL of lysis buffer, split to four series of tubes, heated to 37, 53, 59, or 65°C for 3 mins, and cooled down to 4°C immediately. The cells were lysed by flash-freezing (three cycles) and mechanical shearing with a 30’’ gauge needle 20 times. NP-40 was added to a 0.4% final concentration and incubated with the samples (1 hr, 4°C). The soluble fraction was finally separated by centrifugation (20,000 × *g*, 20 mins).

Each sample (10 µg) was treated with 0.05% Rapigest SF (Waters) and reduced with 20 mM TCEP (tris(2-carboxyethyl)phosphine; 55°C, 20 mins), alkylated with chloroacetamide (Merck) (20°C, 30 mins), and loaded and washed on glass beads using the SP4 protocol (41). The proteins were then digested with 2.33 AU/μL LysC (190 RPM, 37°C, 3 hr) and 0.5 mg/mL trypsin (190 RPM, 37°C, 16 hr). The peptides were subsequently TMT-labeled (6 µg peptides to 3 µL label, 20°C, 16 hrs), desalted with Oasis HLB extraction cartridges (Waters), and demultiplexed using high-pH reverse-phase Zorbax 300 Extend C18 columns (Agilent). The separated peptides were concatenated into 20 fractions and dried. The samples were resuspended in 1% acetonitrile, 0.5% (vol/vol) acetic 6 acid, and 0.06% TFA in water prior to analysis on LC/MS. The spectra were measured with LC-MS2 using liquid chromatography system Dionex 3000 with an Easy-Spray analytical column and Q Exactive HF mass spectrometer over a 70 min gradient of a programmed mixture of mobile phase A (0.1% formic acid) and mobile phase B (99.9% acetonitrile, 0.1% formic acid) to the following gradient over time: 1–55 mins (2%–25%), 55–57 mins (25%–50%), 57–58 mins (50%–85%), 58–63 mins (85%), and 63–70 mins (2%). MS data were acquired using data-dependent acquisition (DDA) with full scan MS spectra acquired in the range of 350–1550 m/z at a resolution of 70,000 and AGC target of 3 × 10^6^; Top12 MS2 35,000 and AGC target of 1 × 10^5^ and 1 × 10^5^ isolation window at 1.2 *m/z*.

### MS-CETSA analysis

The protein abundance was analyzed as described previously (40) and visualized using the R package (https://github.com/SamPazicky/ITDRMS). In short, the raw MS data were analyzed using Proteome Discoverer 2.5 (ThermoFisher Scientific) for peptide and protein identification and quantification, using Mascot 2.6.1 (Matrix Science) using the combined Sanger Institute *P. falciparum* 3D7 coding sequence database and the UniProt database for human proteins. The search parameters used were as follows: MS precursor mass tolerance 30 ppm; MS/MS 0.06 Da; maximal three missed cleavages and static modifications: carbamidomethyl (C); variable modifications: oxidation (M), deamidated (42), acetyl N-terminal protein; forward/decoy searches were used for false discovery rate (FDR) estimation. Peptide spectrum matches (PSMs) with high FDR of 1% and medium FDR of 5% levels were accepted. Abundances relative to control wells were scaled, normalized, and fitted with log-logistic curves. The confidence index was calculated from the fit statistics (*manuscript in preparation*). In short, 95% confidence intervals were calculated for linear fit to 37°C data and log-logistic fits to thermal challenge data. The sum of the confidence intervals (*conf. int _control_ +conf. int _thermal challenge_*) is divided by the increase in the scaled protein abundance compared to the control temperature (*abundance _thermal challenge_ – abundance _control_*). These values are transformed to resemble *P*-values, integrated across each thermal challenge temperature by multiplication, adjusted for multiple testing, and weighted by R^2^ goodness-of-fit values. Negative logarithms of these values are confident indexes, plotted on y-axis on volcano plots against the difference in the area under the dose–response curve (*ΔAUC = AUC _thermal challenge_ – AUC _control_*). The proteins were considered when their confidence index was above log(0.05) and the median absolute deviation of ΔAUC was higher than 3.

### Gene set enrichment analysis

Set enrichment of genes grouped by gene ontology (GO) terms, KEGG pathways (43), and an in-house list of malaria protein complexes was calculated in R (version 4.3.3) (44) using the package fgsea (45). The enriched terms were grouped by hierarchical clustering with complete linkage method using hclust. The dendrogram was plotted using the package ggdendro, and the plot was plotted with ggplot2 and patchwork packages.

### Stage-specific gametocytocidal assays

The stage-specific action of GSK-66 on *P. falciparum* gametocytes was measured in drug assays using a luciferase-based reporter system adapted from previous protocols (46–49). Briefly, gametocytes were induced from the PfNF54-pfs16-GFP-luc reporter line. Drug assays were performed on immature gametocytes (>90% stage II/III) on days 5–6 or on mature gametocytes (>95% stage V) on day 13 at 2% gametocytemia in 1.5% hematocrit cultures. Both immature and mature gametocytes were treated with a 9-point 1:2 dilution series of GSK-66 and azithromycin under hypoxic conditions (90% N_2_, 5% O_2_, and 5% CO_2_) at 37°C. Luciferase activity was subsequently determined in 20 µL of the parasite lysate by adding 50 µL luciferin substrate (Promega Luciferase Assay System) at room temperature, and the resulting bioluminescence was detected using the GloMax- Multi^+^ Detection System with Instinct Software at an integration constant of 10 secs. The IC_50_s were determined on GraphPad Prism with non-linear curve fitting normalized to the maximum and minimum inhibition values, as indicated by the negative vehicle control wells (DMSO). All assays were performed in three independent experiments each with technical replicates.

### Dual gamete formation assays

*P. falciparum* Dual Gamete Formation Assay (PfDGFA) was performed for GSK-66 and azithromycin across 20 concentrations starting at 10 µM, as previously described (50, 51). Briefly, NF54 strain *P. falciparum* mature stage V gametocytes were incubated with dilutions of GSK-66 in 384-well plates for 48 hr at 37 ˚C under 3% (vol/vol) O_2_, 5% CO_2,_ and 92% N_2_. Then, gametogenesis was triggered by reducing the plate temperature to 28 ˚C and adding 100 µM xanthurenic acid and anti-Pf25 (BEI Resources (formerly MR4), cat. no. MRA-315) conjugated to the Cy3 fluorophore. Twenty minutes later, male gametogenesis was recorded by timelapse brightfield microscopy and quantified by computer automated analysis. The plate was then incubated for further 24 hr at 28 ˚C before recording female gametogenesis by fluorescence microscopy of Pfs-25-positive cells and quantification by computer automated analysis. Percent inhibition was calculated with respect to positive and negative controls (1 µM cabamiquine and DMSO, respectively). Assays were performed in quadruplicate replicates with a mean Z-factor of 0.63 ± 0.05 (SD) for the male readout and 0.81  ± 0.08 (SD) for the female readout. IC_50_s were determined from four independent experiments, each conducted with a single technical replicate, using GraphPad Prism by nonlinear regression of a log(inhibitor) vs response – variable slope (four parameters) curve.

## RESULTS

### Azithromycin analogs have potent quick-killing activity against drug-sensitive and resistant *P. falciparum* asexual blood-stage parasites

We (22), and others (24, 29), have previously characterized the quick-killing activity of GSK-66 (or structurally alike compounds) and found using flow cytometry, a 1,615-fold improvement in IC_50_ (0.007 µM) over azithromycin (11.3 µM) with a 44 hr in-cycle treatment against the drug-sensitive D10-PfPHG parasite line (22). In the present study, we recharacterized the quick-killing activity of GSK-66 alongside newly synthesized azithromycin analogs (Fig. 1) in higher-throughput SYBR green-based assays against additional drug-sensitive and -resistant lines (Table 1; Fig. S1). Here, we tested specifically for quick-killing activity over one full asexual blood stage lifecycle to eliminate the apicoplast-mediated delayed-death activity of the potentially dual-acting analogs UAW-1,-2, −3, and −4. All azithromycin analogs were found to have greatly improved quick-killing activity against drug-sensitive 3D7 parasites by 10- to 164-fold over azithromycin (Table 1; Fig. S1a). The most potent growth inhibition was achieved by GSK-66 with an IC_50_ of 0.03 µM, followed by UAW-1 at 0.05 µM. Both analogs contain chloroquinoline modifications and exhibit a 2- to 17-fold increase in potency over the non-chloroquinoline modified analogs, UAW-2 (IC_50_ = 0.11 µM), UAW-3 (IC_50_ = 0.48 µM), and UAW-4 (IC_50_ = 0.14 µM). Among these non-chloroquinoline analogs, UAW-2 with the naphthalene group and UAW-4 with the dichlorophenyl group were significantly more potent than UAW-3 with the phenyl modification. These findings are consistent with previous observations that chemical modification, and specifically the addition of chloroquinoline functional groups, can enhance the quick-killing potential of azithromycin (19, 22).

As the quick-killing activity of azithromycin analogs, particularly those with an added chloroquinoline moiety, has previously been speculated to involve a chloroquine-like mechanism of action (24, 28), we next examined the activity of our analogs on different chloroquine-resistant strains of *P. falciparum* (W2mef, Dd2, 7G8, and Cam3.I) (52, 53). The Cam3.I Cambodian isolate used here also harbors an R539T mutation within the Kelch13 propeller gene, conferring resistance to artemisinin (54). The other *P. falciparum* strains, W2mef, Dd2, and 7G8, are further resistant to pyrimethamine and sulfadoxine, in addition to quinine for Dd2 and mefloquine for W2mef (52). Our results showed that relative to the drug-sensitive 3D7 line, chloroquinoline-containing analogs exhibited no significant shift in IC_50_ in chloroquine-resistant parasites, whereas chloroquine demonstrated a 3.5- to 6-fold increase (Table 1; Fig. S1b). In contrast, azithromycin and analogs with a non-chloroquinoline moiety (UAW-2,-3, and −4) tended to be equally or more potent in multidrug-resistant parasites (Table 1; Fig. S1b). Overall, these and previous data (22, 24, 29) suggest that azithromycin analogs, including those with chloroquinoline modifications, maintain useful activity against chloroquine-resistant parasites over 72 hrs of treatment.

### Verapamil, a PfCRT inhibitor, sensitizes chloroquine-resistant Dd2 parasites to chloroquinoline but not non-chloroquinoline-containing azithromycin analogs

Chloroquine resistance is attributed to mutations in the *P. falciparum* chloroquine resistance transporter (PfCRT), which facilitates efflux of chloroquine away from its site of action in the parasite’s digestive vacuole. Verapamil is known to work in synergy with chloroquine by inhibiting these mutant isoforms of the PfCRT, thereby allowing chloroquine to maintain its activity in chloroquine-resistant parasites (55–57). Following previous observations in chloroquine-resistant *P. falciparum* lines (Table 1; Fig. S1b), we next sought to investigate whether our azithromycin analogs are susceptible to the same drug efflux mechanism through the PfCRT in drug synergy assays with verapamil (33). In these assays, we treated malaria parasites with different ratios of the drug combination to determine the fractional inhibitory concentrations (FIC_50_). Isobologram curves comparing the FIC_50_ values were used alongside the mean ΣFIC_50_ to interpret the drug interaction profile. Concave curves below the dotted additivity reference line generally indicate an interaction tending toward synergy, whereas convex curves above the additivity reference indicate an interaction tending toward antagonism. To minimize the impact of experimental variation on synergy and antagonism interpretations, drug combinations exhibiting a mean ΣFIC_50_ <0.8 were considered synergistic, drug combinations exhibiting a mean FIC_50_ ≥1.4 were antagonistic, and if equal to 0.8–1.4 were additive (34–36).

**Fig 2:**
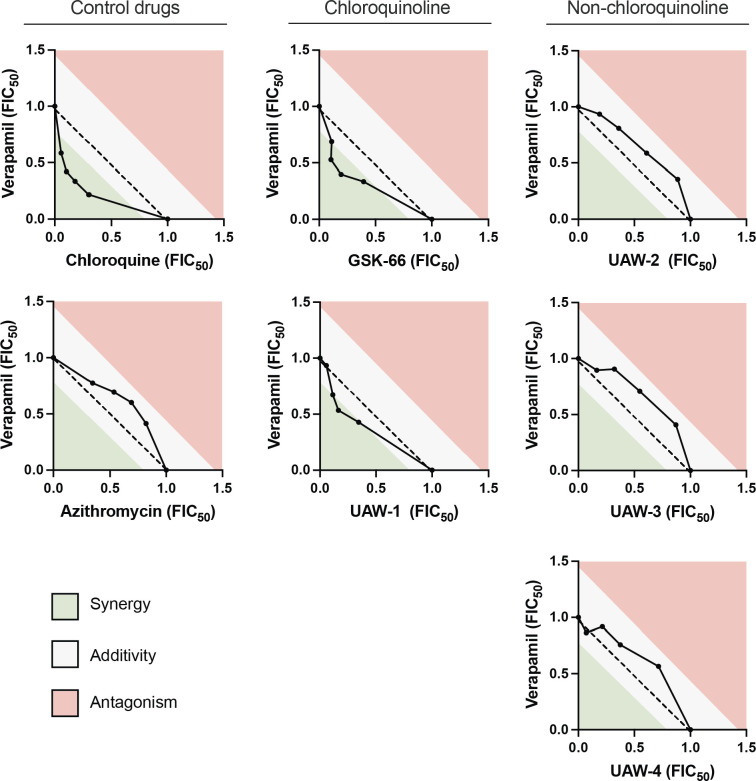
Interaction between drug efflux inhibitor verapamil and azithromycin analogs in synergy assays with chloroquine-resistant *P. falciparum* Dd2 parasites. Dd2 parasites were treated with different concentration ratios of each drug combination, and parasite growth was subsequently measured for three or more independent experiments to calculate the fractional inhibitory concentration (FIC_50_). The concavity of the isobolograms was used alongside the mean ΣFIC_50_s in Table 2 to interpret the interaction between drugs. Concave curves below the dotted additivity reference line generally indicate synergy, and convex curves above the additivity reference point towards antagonism.

Using chloroquine-resistant Dd2 parasites, we found that the combination of verapamil and chloroquine was associated with a concave isobologram curve and a mean ΣFIC_50_ of 0.5 in our assays, indicating a strong synergistic effect, as expected. We tested for resistance reversal to the unmodified azithromycin parent compound and demonstrated that azithromycin in combination with verapamil produces a weakly convex curve and a mean ΣFIC_50_ of 1.2 (Fig. 2; Table 2). This represents an additive effect in the direction of antagonism, where the two drugs are likely working independently of each other. We next tested for resistance reversal to the chloroquinoline-containing azithromycin analogs, GSK-66 and UAW-1. The combined treatment of verapamil with GSK-66 resulted in a concave isobologram curve and mean ΣFIC_50_ of 0.7, indicating a synergistic interaction (Fig. 2; Table 2). The mean ΣFIC_50_ for verapamil and UAW-1 of 0.8 falls just within the 0.8–1.4 range for additivity (Table 2); however, when considered with the chloroquine-like concave isobologram curve, we interpreted this interaction as tending toward synergy rather than a true additive effect (Fig. 2). In contrast, the non-chloroquinoline-containing analogs, UAW-2,-3, and −4, all demonstrated an additive effect in the direction of antagonism, with weakly convex isobologram curves and mean ΣFIC_50_s between 0.8 and 1.4 (Fig. 2; Table 2). These data indicate that the azithromycin azalide scaffold and non-chloroquinoline-containing analogs are not susceptible to verapamil-induced reversal of chloroquine resistance, but the chloroquinoline-containing analogs show weak susceptibility.

**TABLE 2:** Fractional inhibitory concentrations from treatment of chloroquine-resistant Pf Dd2 parasites for fixed-ratio combinations of verapamil (VPM) with chloroquine (CQ), azithromycin (AZR), or azithromycin analogs^*a*^

Drug combination	ΣFIC_50_ (±SEM)				Mean ΣFIC_50_ (±SEM)	Interaction
	4:1	3:2	2:3	1:4		
CQ +VPM	0.5 (0.04)	0.5 (0.1)	0.5 (0.1)	0.6 (0.2)	0.5 (0.1)	Synergistic
AZR +VPM	1.2 (0.2)	1.3 (0.2)	1.2 (0.04)	1.1 (0.07)	1.2 (0.1)	Additive
GSK-66 + VPM	0.7 (0.1)	0.6 (0.2)	0.6 (0.3)	0.8 (0.2)	0.7 (0.2)	Synergistic
UAW-1 +VPM	0.8 (0.07)	0.7 (0.2)	0.8 (0.2)	1.0 (0.07)	0.8 (0.1)	Additive
UAW-2 +VPM	1.2 (0.1)	1.2 (0.07)	1.2 (0.2)	1.2 (0.1)	1.2 (0.1)	Additive
UAW-3 +VPM	1.3 (0.04)	1.3 (0.1)	1.2 (0.2)	1.1 (0.04)	1.2 (0.1)	Additive
UAW-4 +VPM	1.3 (0.1)	1.1 (0.08)	1.1 (0.3)	0.9 (0.03)	1.1 (0.1)	Additive

^*a*^ Data represent mean ± SEM, *n* ≥ 3.

### Azithromycin analogs have quick-killing activity resembling chloroquine’s inhibition of hemoglobin digestion in synergy assays with the cysteine protease inhibitor, E64

Chloroquine, along with other 4-aminoquinolines, is most widely accepted to exert its anti-malarial activity in the parasite’s digestive vacuole by inhibiting the polymerization of toxic heme products from hemoglobin degradation into a non-toxic crystalline form known as hemozoin (58–60). In addition to this mechanism, chloroquine has also been implicated in the disruption of hemoglobin digestion, potentially through the alteration of vacuolar pH (61), or by inhibiting the activity of proteases responsible for hemoglobin hydrolysis (62).

**Fig 3:**
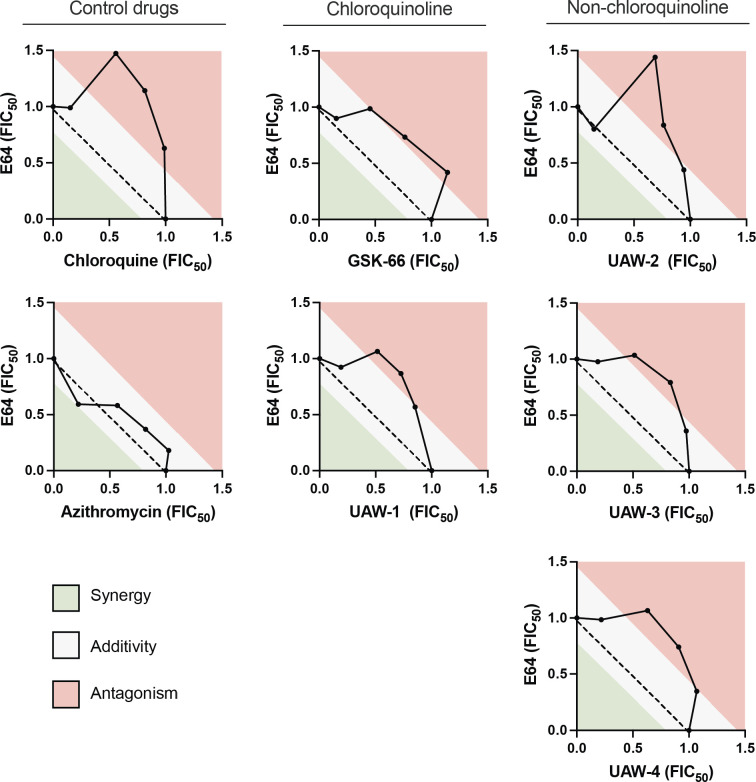
Interaction between hemoglobin digestion inhibitor, E64, and azithromycin analogs in synergy assays with drug-sensitive *P. falciparum* 3D7 parasites. 3D7 parasites were treated with different concentration ratios of each drug combination, and parasite growth was subsequently measured for three or more independent experiments to calculate the FIC_50_. The concavity of the isobolograms was used alongside the mean ΣFIC_50_s in Table 3 to interpret the interaction between drugs.

To investigate whether our azithromycin analogs act through the same mechanisms as chloroquine in the parasite’s digestive vacuole, we next undertook a series of synergy assays using compounds known to interfere with hemoglobin degradation pathways. E64 is an inhibitor of cysteine proteases, such as falcipains, which play an important role in the hydrolysis of hemoglobin (63, 64). E64 has previously been shown to antagonize the activity of chloroquine in *in vitro* synergy assays, presumably by reducing the amount of toxic heme products available for chloroquine binding (65). As expected, in our synergy assays using drug-sensitive 3D7 parasites, we also observed a strong antagonism for E64 and chloroquine, with a convex isobologram curve and a mean ΣFIC_50_ of 1.7 (Fig. 3; Table 3).

**TABLE 3:** Fractional inhibitory concentrations from treatment of chloroquine-sensitive Pf 3D7 parasites with fixed-ratio combinations of E64 with chloroquine (CQ), azithromycin (AZR), or azithromycin analogs^*a*^

Drug combination	ΣFIC_50_ (±SEM)				Mean ΣFIC_50_ (±SEM)	Interaction
	4:1	3:2	2:3	1:4		
CQ +E64	1.6 (0.3)	2.0 (0.1)	2.0 (0.6)	1.1 (0.3)	1.7 (0.07)	Antagonistic
AZR +E64	1.2 (0.04)	1.2 (0.09)	1.1 (0.05)	0.8 (0.06)	1.1 (0.05)	Additive
GSK-66 + E64	1.6 (0.05)	1.5 (0.2)	1.4 (0.07)	1.1 (0.05)	1.4 (0.06)	Antagonistic
UAW-1 +E64	1.4 (0.2)	1.6 (0.07)	1.6 (0.1)	1.1 (0.1)	1.4 (0.05)	Antagonistic
UAW-2 +E64	1.4 (0.05)	1.5 (0.03)	1.5 (0.2)	1.0 (0.1)	1.4 (0.04)	Antagonistic
UAW-3 +E64	1.3 (0.06)	1.6 (0.08)	1.5 (0.07)	1.2 (0.07)	1.4 (0.03)	Antagonistic
UAW-4 +E64	1.4 (0.03)	1.6 (0.08)	1.7 (0.1)	1.2 (0.2)	1.5 (0.06)	Antagonistic

^*a*^ Data represent mean ± SEM, *n* ≥ 3.

For both the chloroquinoline and non-chloroquinoline azithromycin analogs, their interactions with E64 were associated with convex isobologram curves and mean ΣFIC_50_s of ≥1.4, pointing toward a similar antagonistic trend, as observed for chloroquine (Fig. 3; Table 3). For the azithromycin parent compound, we saw only a small additive effect, indicating that the hemoglobin digestion inhibitor has minimal impact on the quick-killing activity of azithromycin. These data suggest that the quick-killing activity of both chloroquinoline and non-chloroquinoline azithromycin analogs is sensitive to disruption of hemoglobin digestion, as also occurs for chloroquine.

### Azithromycin analogs have quick-killing activity resembling chloroquine’s inhibition of hemozoin formation in synergy assays with the iron chelator, desferrioxamine

**Fig 4:**
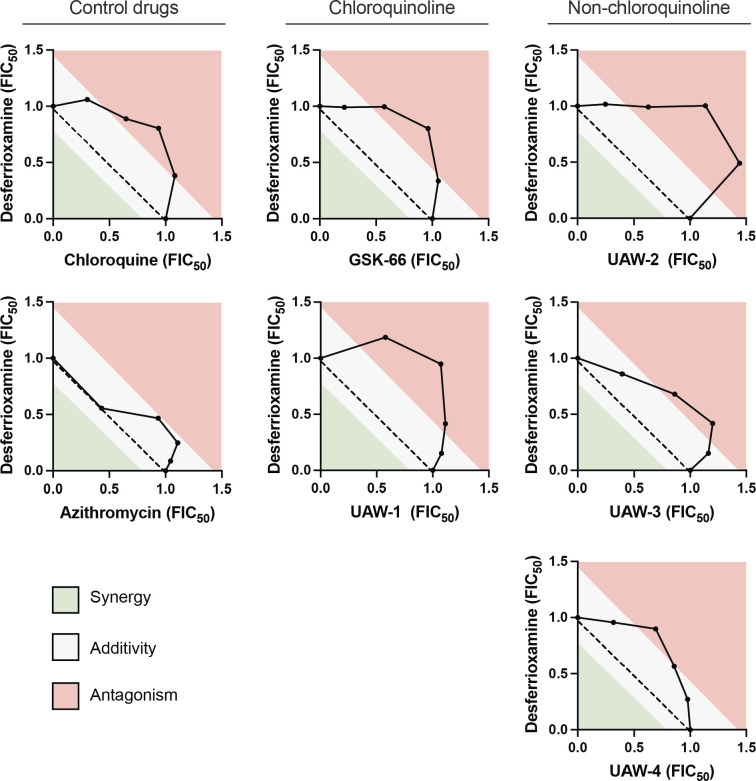
Interaction between hemoglobin digestion inhibitor, desferrioxamine, and azithromycin analogs in synergy assays with drug-sensitive *P. falciparum* 3D7 parasites. 3D7 parasites were treated with different concentration ratios of each drug combination, and parasite growth was subsequently measured for three or more independent experiments to calculate the FIC_50_. The concavity of the isobolograms was used alongside the mean ΣFIC_50_s in Table 4 to interpret the interaction between drugs.

The iron chelator desferrioxamine (DFO) has been shown in *in vitro* synergy assays to counteract chloroquine’s inhibitory effect against heme polymerization (66, 67). While the exact mechanism of action remains unknown, DFO is postulated to bind hematin, an oxidized form of heme, and accelerate its polymerization into hemozoin (68). In our synergy assays using drug-sensitive 3D7 parasites, we also observed an antagonistic interaction between DFO and chloroquine, as demonstrated by a convex isobologram curve and a mean ΣFIC_50_ of 1.5 (Fig. 4; Table 4).

**TABLE 4:** Combinatory effect of fixed-ratio drug combinations with desferrioxamine (DFO) against chloroquine-sensitive Pf 3D7 strain^*a*^

Drug combination	ΣFIC_50_ (±SEM)				Mean ΣFIC_50_ (±SEM)	Interaction
	4:1	3:2	2:3	1:4		
CQ +DFO	1.5 (0.1)	1.7 (0.1)	1.5 (0.3)	1.4 (0.2)	1.5 (0.14)	Antagonistic
AZR +DFO	1.1 (0.06)	1.4 (0.03)	1.4 (0.1)	1.0 (0.06)	1.2 (0.05)	Additive
GSK-66 + DFO	1.4 (0.1)	1.8 (0.2)	1.6 (0.1)	1.2 (0.1)	1.5 (0.14)	Antagonistic
UAW-1 +DFO	1.2 (0.1)	1.5 (0.2)	2.0 (0.4)	1.8 (0.3)	1.6 (0.16)	Antagonistic
UAW-2 +DFO	1.9 (0.3)	2.1 (0.4)	1.6 (0.2)	1.3 (0.2)	1.7 (0.08)	Antagonistic
UAW-3 +DFO	1.3 (0.09)	1.6 (0.05)	1.5 (0.08)	1.3 (0.1)	1.4 (0.04)	Antagonistic
UAW-4 +DFO	1.2 (0.1)	1.4 (0.06)	1.6 (0.06)	1.3 (0.2)	1.4 (0.01)	Antagonistic

^*a*^ Data represent mean ± SEM, *n* ≥ 3.

We next tested for susceptibility of our azithromycin analogs to DFO’s reversal of heme polymerization inhibition. In these experiments, we found that both chloroquinoline and non-chloroquinoline azithromycin analogs were associated with convex isobologram curves and mean ΣFIC_50_s of ≥1.4, indicating an antagonistic interaction like chloroquine (Fig. 4; Table 4). On the other hand, for the azithromycin parent compound, we saw only a small additive effect in the direction of antagonism (Fig. 4; Table 4). This suggests that the quick-killing activity of azithromycin itself is unaffected by DFO’s chelation of iron. Therefore, for both chloroquinoline and non-chloroquinoline-containing azithromycin analogs, these results point toward a mechanism of quick-killing that involves a chloroquine-like inhibition of hemozoin formation.

### Chloroquinoline, but not non-chloroquinoline analogs, inhibits β-hematin polymerization in cell-free assays

Previously, Peric et al. (29) reported that azithromycin analogs containing chloroquinoline and quinoline modifications, but not a naphthalene modification, could inhibit heme polymerization in cell-free assays like chloroquine does (29). In contrast, we found that in whole-cell assays, neither the chloroquinoline-modified analog, GSK-66, nor a quinoline-containing analog fully recapitulated the chloroquine-like impact on heme and hemozoin levels (22). Since we observed that DFO antagonizes the quick-killing activity of both chloroquinoline and non-chloroquinoline analogs in live cells, we next wanted to investigate whether they could directly inhibit β-hematin polymerization in cell-free assays.

**Fig 5:**
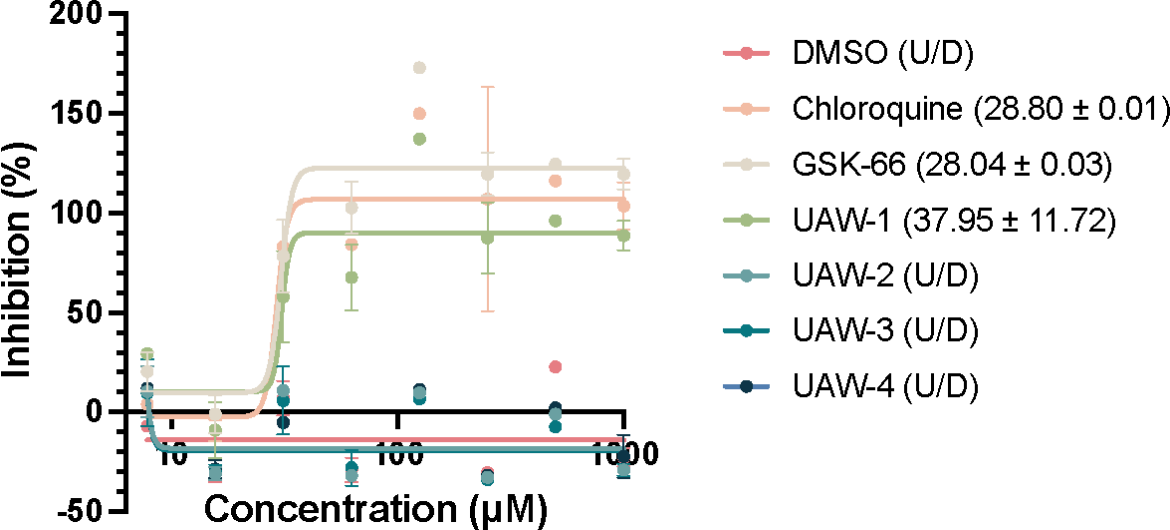
Inhibition of β-hematin polymerization by chloroquinoline and non-chloroquinoline-containing drugs in an *in vitro* propionate buffer system. Drug inhibition of the chemical conversion from heme to β-hematin was determined by measuring the absorbance of non-converted heme after a 16 h compound incubation relative to controls. The mean IC_50_s for each drug are presented in the brackets (mean IC_50_ ±SEM, μM), and compounds with no determined IC_50_ values at the concentrations used in this experiment (U/D = undefined) were considered non-inhibitory. Error bars represent the SEM for two independent experiments, performed in technical replicates or triplicates.

Our results showed that the chloroquinoline-containing analogs GSK-66 and UAW-1 inhibited formation of β-hematin at a similar efficiency as chloroquine (Fig. 5). The non-chloroquinoline-containing analogs, UAW-2,-3, and −4, demonstrated no inhibition at the concentrations tested, matching that of the DMSO control (Fig. 5). These results contradict the synergy assays with DFO for the non-chloroquinoline azithromycin analogs, but support a chloroquine-like inhibition of β-hematin formation for the quick-killing activity of chloroquinoline-containing analogs.

### Resistance to the quick-killing activity of GSK-66 is unable to be generated *in vitro*

We next investigated alternative mechanisms of quick-killing activity that may be prominent in azithromycin analogs by attempting to generate drug-resistant parasites *in vitro* to our most potent analog, GSK-66. Previous attempts to generate resistance to quick-killing azithromycin analogs in a standard laboratory line (D10) had been unsuccessful (22). Therefore, we sought to potentiate resistance selection using a Dd2 clone that harbors mutations in the parasite’s DNA polymerase δ and elevates the mutation accumulation rate in drug-pressured lines by 13- to 28-fold (38). As this Dd2 clone still possesses a functional apicoplast, we only attempted to select for drug resistance using GSK-66, where the modification on the desosaminyl carbohydrate is expected to remove apicoplast-targeting delayed-death activity. Two attempts to select for resistant parasites via cyclic treatment with 10 × IC_50_ of GSK-66 did not produce recrudescent parasites. Repeated attempts for resistance selection using a lower drug pressure at 2 × IC_50_ of GSK-66 produced viable parasites; however, subsequent drug inhibition assays revealed a non-significant change in IC_50_ by 1.2-fold relative to the control DMSO-treated population (Fig. S2). Additional resistance selection of these parasites with GSK-66 treatment at 3 × IC_50_ did not produce any recrudescent parasites for more than 1 month following drug removal. These findings suggest that the quick-killing mechanism of action of GSK-66 is one to which resistance is difficult to generate *in vitro*.

### Cellular thermal shift proteomics demonstrates that azithromycin analogs significantly stabilize a broad range of parasite proteins

As we were unable to generate drug-resistant parasites, we attempted to identify potential non-apicoplast azithromycin analog drug targets using Cellular Thermal Shift Assays (CETSA). In these experiments, we exposed either lysed or live *P. falciparum* 3D7 trophozoites to increasing doses of the drug, precipitated the proteome at four fixed temperatures, and used mass spectrometry to quantify the soluble protein fraction. Increasing thermal stabilization of specific proteins with the increasing drug dose in parasite lysates represents direct protein-drug binding, whereas the thermal stabilization in intact cells can additionally indicate other drug-induced effects related to the mechanism of action, including protein interactions with other proteins, metabolites, or even post-translational modifications (40, 62). Here, we tested three of our most potent azithromycin analogs, GSK-66, UAW-1, and UAW-2. We reasoned that the two chloroquinoline-containing drugs, GSK-66 and UAW-1, would allow for the identification of a shared chloroquinoline-associated mechanism of quick-killing, whereas UAW-2 (structurally equivalent to UAW-1) protein stabilization may reveal similarities and differences between chloroquinoline and non-chloroquinoline analog activity.

Performing CETSA on lysate samples exposed to GSK-66, UAW-1, and UAW-2 each yielded a distinct set of hits (Fig. S3a). While significantly thermal stabilized, these proteins, however, displayed either mixed destabilizing and stabilizing curves at different temperatures or a low magnitude of response (<1.3) (Fig. S3b). Moreover, none of them were thermally stabilized in the intact-cell CETSA and therefore were not explored further.

**Fig 6:**
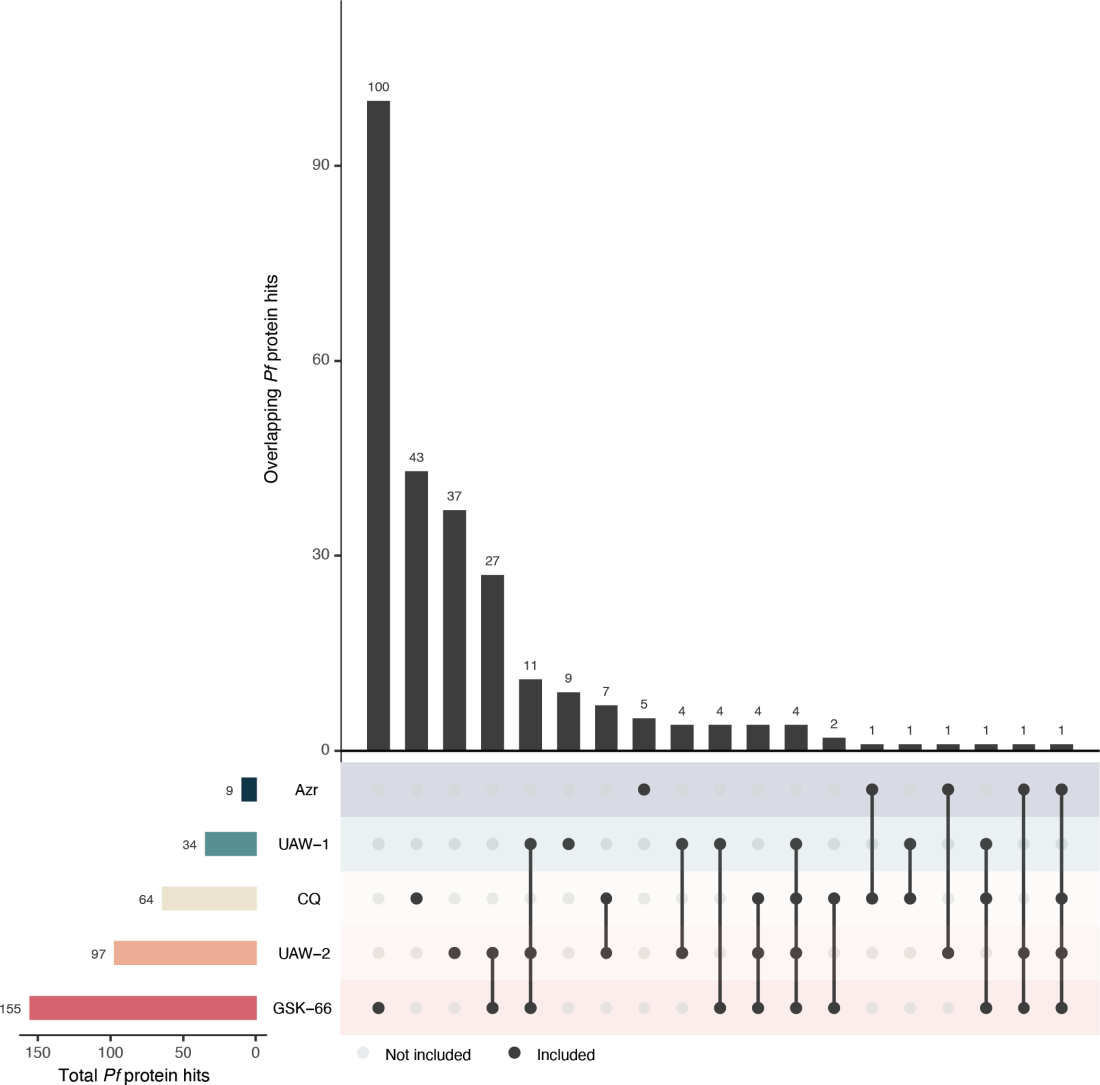
Shared and unique *P. falciparum* proteins identified by intact-cell CETSA as significantly thermal stabilized. Pf 3D7 trophozoites were treated with chloroquine (CQ), azithromycin (Azr), or azithromycin analogs before cell lysis for identification of significantly stabilized proteins. All experiments were conducted with at least one biological replicate.

For the intact-cell experiments, we solubilized the treated parasites in NP-40 for processing, which can lead to increased proteome coverage by including membrane proteins that would typically be excluded from the soluble fraction. In these experiments, we observed high levels of significantly stabilized proteins for most compounds, with 18.5% of all detected parasite proteins being associated with the membrane (GO:0016021 and/or GO:0016020). Treatment with the chloroquinoline-containing GSK-66 resulted in the greatest response, yielding 155 significantly stabilized *P. falciparum* protein hits, followed by the naphthalene-containing UAW-2 with 97 hits, chloroquine with 64 hits, the other chloroquinoline-containing UAW-1 with 34 hits, and finally azithromycin with nine hits (Fig. 6). These numbers generally exceed what has previously been reported in the literature for other anti-malarials, including chloroquine, and for compounds targeting human cells, which have a much larger detectable proteome (62, 69), highlighting the improved proteomic coverage of the intact-cell CETSA protocol applied here. However, even with these higher detection levels, GSK-66-treated parasites still overwhelmingly demonstrated the greatest impact on parasite protein stabilization upon drug treatment.

From the identified hits in the intact-cell data (Fig. S4a), we observed some weak trends between azithromycin, chloroquine, and the azithromycin analogs. First, the chloroquinoline-containing analogs demonstrated minimal overlap in significantly stabilized proteins with chloroquine. Of all hits, only the 26S proteasome regulatory subunit RPN7 (PF3D7_1129200) was exclusively shared by chloroquine and the chloroquinoline-containing azithromycin analogs, GSK-66 and UAW-1. Additionally, despite sharing the same backbone, there were no protein hits commonly stabilized by azithromycin and the three analogs. This trend also encompassed proteins of the apicoplast 50 s ribosome, the known target of azithromycin, where there was no evidence of stabilized proteins for this complex in either the lysate or intact-cell CETSA for both azithromycin and its analogs (Fig. S5 and S6). For falcilysin (PF3D7_1360800), a previously identified CETSA hit for chloroquine (62, 70), we observed low levels of stabilization with chloroquine treatment (ΔAUC = 0.89), which was broadly similar across the azithromycin analogs, GSK-66 (1.11), UAW-1 (1.71), and UAW-2 (1.21), but less so for azithromycin (0.14) (Fig. S4b). However, in our experiment, stabilization of falcilysin was less apparent than for other proteins significantly stabilized by these drugs.

The strongest and most convincing similarities were between the three quick-killing azithromycin analogs, GSK-66, UAW-1, and UAW-2. Across these, there were 11 commonly stabilized protein hits, including several with annotations relating to gene regulation, such as a regulator of chromosome condensation (PF3D7_0711500), the serine-/arginine-rich splicing factor 12 (PF3D7_0503300) and three translation initiation factors (PF3D7_0607000, PF3D7_1206200, and PF3D7_1212700). Two of the 11 proteins were molecular chaperones or co-chaperones, including the heat shock protein 110 (PF3D7_0708800) and Hsp70/Hsp90 organising protein (PF3D7_1434300). Other shared hits included a putative ubiquitin-specific protease (PF3D7_0904600), the RING finger E3 ubiquitin-protein ligase (PF3D7_1004300), a CUGBP Elav-like family member 1 (PF3D7_1359400), and the putative vacuolar fusion protein MON1 (PF3D7_1352800). Four other proteins were commonly stabilized between the two chloroquinoline-containing azithromycin analogs GSK-66 and UAW-1, but not with other drug treatments, including an ATPase and activator of Hsp90 (PF3D7_0306200), two RNA-binding proteins (PF3D7_0610200 and PF3D7_0823200), and elongation factor 1-beta (PF3D7_0913200). Overall, the majority of the thermal stabilized proteins were unique to each compound (Fig. 6).

**Fig 7:**
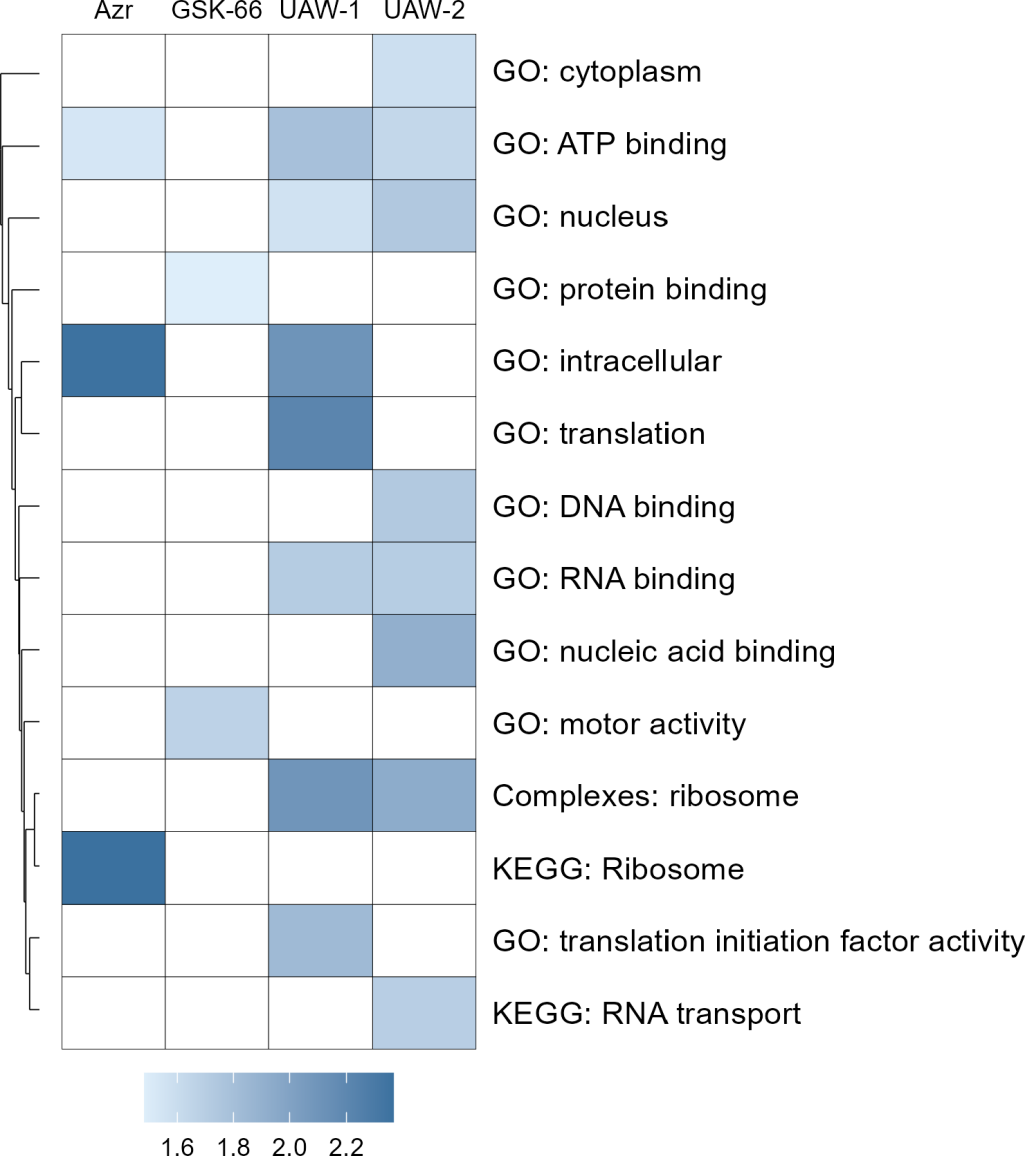
Gene set enrichment analysis of parasite protein hits from intact cell CETSA based on Gene Ontology (GO) terms and KEGG pathways. Pathways were considered enriched when a statistically significant proportion of genes within a gene set showed significant thermal stabilization in the intact cell CETSA. Dark blue indicates more strongly enriched pathways or GO terms, while light blue shows weaker enrichment. GO terms and KEGG pathways are clustered based on the overlap of genes in each set, as represented by the dendrogram.

Due to the large number of target proteins identified for each of the azithromycin analogs, we used gene set enrichment analysis (GSEA) to identify the cellular processes that were overrepresented by all thermal-shifted proteins in the data set (Fig. 7). Pathways were considered enriched when a statistically significant proportion of genes within a gene set (*P* < 0.05) exhibited thermal stabilization in the intact-cell CETSA. For parasites treated with chloroquine, we did not observe significant enrichment of any Gene Ontology or KEGG pathway terms. In contrast, treatment with azithromycin led to enrichment of intracellular pathways, primarily associated with the ribosome and ATP-binding proteins (Fig. 7). The enriched ribosome-associated proteins are mostly components of cytoplasmic ribosomes (Fig. S7) and not apicoplast ribosomes, where we failed to confidently detect any stabilized proteins (Fig. S5 and S6). The GSEA for UAW-1 and UAW-2 demonstrated some overlap with the enrichment profile of azithromycin (UAW-1 = ATP binding, intracellular and ribosome; UAW-2 = ATP binding and ribosome), with a particular focus on translation-related terms for UAW-1, including translation and translation initiation factor activity. However, unlike azithromycin, UAW-1 and UAW-2 also enriched for pathways linked to activity in the nucleus, such as RNA binding, and DNA binding for UAW-2, suggesting a broader impact on gene regulation for these analogs. GSK-66, on the other hand, primarily led to detectable stabilization of proteins involved in protein binding and motor activity, differentiating its drug response from that of chloroquine, azithromycin, and the other analogs with this technique. While the CETSA data were unable to reveal a likely shared mechanism of action for these quick-killing azithromycin analogs, they provide valuable insights into the broad range of potential cellular targets impacted by these drugs.

### Lead compound GSK-66 shows activity against sexual stage gametocytes

Having identified several azithromycin analogs with significantly improved quick-killing activity against asexual blood-stage parasites, we next assessed the transmission-blocking potential of our most potent analog, GSK-66. Currently, most clinically used anti-malarials that have activity against immature (stage I–III) gametocytes, including chloroquine (71) and artemisinin (72), are considered ineffective against late-stage (stage IV–V) gametocytes. These mature gametocytes are the only stage functionally capable of transmission back to the mosquito for further human infection (48, 73). To determine whether GSK-66 is suitable as a transmission-blocking anti-malarial, we first evaluated its gametocytocidal activity against immature (II–III) and mature (>95% stage V) gametocyte populations in 48 hr assays measuring luciferase activity. The results showed that GSK-66 has significantly improved activity over its parent compound azithromycin by 167-fold against immature gametocytes (GSK-66 IC_50_ = 0.06 µM; azithromycin IC_50_ >10 µM) and 7-fold against mature gametocytes (GSK-66 IC_50_ = 1.48 µM; azithromycin IC_50_ >10 µM) (Table 5; Fig. S8). The activity of GSK-66 against the mature stages was approximately 24-fold lower compared to the immature stages. It was previously reported by Reader et al. that chloroquine has an IC_50_ of 0.006 µM on immature gametocytes in the same assay platform; however, it is >848 fold less potent against mature gametocytes (IC_50_ >5 µM) (48). Therefore, our data demonstrate that modifications made to azithromycin through GSK-66 can greatly improve activity against gametocyte stages, exceeding the late-stage gametocyte killing potential of chloroquine.

**TABLE 5:** Activity of lead compound GSK-66 against different stages of gametocyte development in 48 hr assays for GSK-66^*a*^

Stage of gametocyte activity	IC_50_ (± SEM) μM	
	GSK-66	Azithromycin
Immature stage II–III	0.06 (0.01)	>10 ^*b*^
Mature stage V	1.48 (0.26)	>10^*b*^
Male gamete formation	0.28 (0.01)	12.62 (0.92)
Female gamete formation	0.44 (0.05)	28.60 (0.95)

^*a*^ Data represent mean ± SEM, *n* ≥ 3.

^*b*^ IC_50_ not determined as the highest concentration tested was 10 µM.

It was previously noted that male gametocytes, which are more sensitive to anti-malarial drugs than female gametocytes (74), may only represent 25% of the total gametocyte population. Hence, compounds with male-targeted activity tend to be overlooked in gametocyte killing assays (73, 75). To determine whether GSK-66 may have sex-specific activity against late-stage gametocytes, we also measured its ability to inhibit formation of male and female gametes *in vitro* using Dual Gamete Formation Assays. Consistent with previous findings (74, 76), azithromycin demonstrated modest inhibition of male and female gametes with IC_50_s of 12.62 µM and 28.60 µM, respectively (Table 5; Fig. S9). Comparatively, GSK-66 was 45- to 65-fold more potent than azithromycin, inhibiting the formation of male gametes at an IC_50_ of 0.28 µM and of female gametes at an IC_50_ of 0.44 µM (Table 5). This is in contrast to other drugs, such as pyrimethamine and the artemisinins, where activity against female gametocytes is reported to be negligible (74). These data indicate that GSK-66 could be developed as a fast, potent, and broad-acting drug against blood-stage parasites that also has potential transmission-blocking activity.

## DISCUSSION

The emergence of clinical resistance to nearly all currently available anti-malarials, including our most critical family of artemisinins, highlights the urgent need for new drugs with novel mechanisms of action to curb the declining efficacy of malaria treatment strategies (4, 5, 77). While azithromycin offers several favorable pharmacokinetic and safety characteristics for repurposing as an anti-malarial, its use for preventing and treating clinical cases of malaria remains suboptimal compared to existing anti-malarial drugs (78). The recent discovery that azithromycin can be chemically modified to impart potent activity through additional quick-killing mechanisms highlights the promising potential to improve the drug’s therapeutic properties (19, 22). However, the mechanism by which this quick-killing occurs remains to be elucidated, with evidence suggesting a chloroquine-like mechanism of action may contribute for some analogs (24, 28). Here, we investigated the quick-killing activity of five azithromycin analogs and demonstrated that both chloroquinoline and non-chloroquinoline-containing analogs exhibit anti-malarial activity resembling chloroquine’s inhibition of heme polymerization and hemoglobin digestion. However, the mechanism of action for these quick-killing compounds is likely to encompass a much broader range of potential targets, causing disruption of many pathways that are different from chloroquine.

Consistent with previous studies (22, 28, 29), we observed that while both chloroquinoline and non-chloroquinoline modifications could improve quick-killing activity over azithromycin by 9- to 160-fold, those with added chloroquinoline groups exhibited a greater improvement in quick-killing potency of 100- to 160-fold over the parent compound azithromycin. One of the key questions investigated in this study was the role of the chloroquinoline moiety in the mechanism of action of azithromycin analogs that contained this functional group. The potential for azithromycin analogs with a chloroquinoline moiety to act predominantly through a chloroquine-like mechanism has previously been speculated, but whether this occurs biologically within the parasite is yet to be resolved (24, 28, 29).

As previously reported (29), we also found that chloroquinoline-containing azithromycin analogs specifically inhibit the formation of β-hematin in cell-free assays similarly to chloroquine, but this was not observed for non-chloroquinoline-containing analogs. Paradoxically, we have previously shown the absence of any clear chloroquine-like activity against hemoglobin digestion and hemozoin formation in live cells for our lead chloroquinoline analog, GSK-66, whereas the quinoline-containing GSK-71 did exhibit a measurable inhibition of hemoglobin digestion (22). In the present study, we observed that chemical inhibition of hemoglobin digestion and chloroquine’s inhibitory effect against heme polymerization reduces the potency of chloroquinoline-containing analogs, as seen for chloroquine. This property, however, was also shared with non-chloroquinoline-containing analogs, supporting a general link between parasite feeding and the mechanism of action of quick-killing azithromycin analogs with both types of structural moieties and independent of the azithromycin backbone.

Additionally, when we explored changes in protein thermal stabilization upon drug treatment in the intact-cell CETSA, we found minimal overlap in significantly stabilized proteins between chloroquine and analogs containing the chloroquinoline moiety. In fact, the strongest similarities were by far between the three quick-killing azithromycin analogs, with 11 commonly stabilized protein hits compared to just one for chloroquine and the chloroquinoline-containing analogs, GSK-66 and UAW-1. Taking into consideration all changes in parasite protein homeostasis in the intact cell CETSA, the GSEA supported enrichment of proteins across similar pathways for UAW-1 and UAW-2. These proteins were primarily associated with gene regulation, whereas GSK-66 treatment enriched for proteins involved in protein binding and motor activity. In both cases, these pathways and terms were not enriched for by chloroquine. For falcilysin, a known protein hit of chloroquine in CETSA experiments (62, 70), we also observed stabilization with chloroquine treatment in our study. Although the relative thermal shift in falcilysin between untreated and chloroquine-treated parasites did not exceed our criteria to be considered a stabilized hit, our azithromycin analogs, but not azithromycin, exhibited broadly similar, if not higher, levels of stabilization of falcilysin over chloroquine. As previously reported by Wirjanata et al. (62), the inhibitory site of falcilysin where chloroquine binds is promiscuous to binding by other quinoline drugs and compounds with unrelated structures like MK4815, MMV000848, or MMV665806. Therefore, there remains the possibility that our azithromycin analogs can bind falcilysin; however, it appears likely that they bind more significantly to a range of other proteins. It is interesting to note that a previous screen for β-hematin inhibitors using a cell-free system identified >500 hits, with representative drugs from the 14 primary scaffolds identified all demonstrating inhibitory activity against parasite hemozoin formation (79), suggesting that many chemical scaffolds had at least background activity that resembled chloroquine’s mechanism of action. Therefore, while we and others have found evidence that azithromycin analogs exhibit properties similar to chloroquine, these results do not present clear evidence that a chloroquine-like mechanism of action is the dominant pathway of parasite killing for either the chloroquinoline or non-chloroquinoline azithromycin analogs.

From a practical standpoint, an important question is as follows: what is the potential impact of established parasite chloroquine resistance on azithromycin analog activity? In this study, chloroquine-resistant parasites showed limited resistance to chloroquinoline-containing analogs, whereas non-chloroquinoline analogs were generally equipotent or exhibited an increase in potency. Previous studies have identified similar trends with chloroquinoline and non-chloroquinoline-containing quick-killing azithromycin analogs showing minimal to no loss of efficacy against chloroquine-resistant parasites (22, 29). In the present study, however, we also showed that the chloroquine resistance reversal agent, verapamil, is capable of sensitizing chloroquine-resistant Dd2 parasites to chloroquinoline-containing analogs, pointing toward a shared susceptibility to drug efflux through the PfCRT. However, it was apparent that synergy with verapamil was less pronounced for chloroquinoline-containing azithromycin analogs than seen for chloroquine, suggesting that overall, chloroquinoline-containing azithromycin analogs have some resilience against typical measures of chloroquine resistance. We speculate that the larger size and differential charge structure of the chloroquinoline-containing azithromycin analogs could potentially contribute to this resilience. A previous study identified that piperaquine-resistant isoforms of PfCRT harbor additional mutations within the drug-interacting central cavity, potentially to accommodate for interactions with a larger and more positively charged drug than chloroquine (80). Given that the azithromycin analogs used in this study are much larger, >1.5 times the molecular weight of chloroquine or piperaquine, it is possible that this increased size may limit their binding or export through the Dd2 PfCRT and other resistance isoforms, resulting in reduced effectiveness of PfCRT mutations and drug efflux. Therefore, these chloroquinoline-containing azithromycin analogs maintain useful activity against chloroquine-resistant parasites.

Several lines of evidence presented in our study support the contribution of other non-chloroquine-related mechanisms to quick-killing. First, quick-killing activity is observed for azithromycin itself, despite not featuring a chloroquine-like functional group or exhibiting inhibitory activity against hemoglobin digestion and heme polymerization as chloroquine does in synergy assays. Second, azithromycin analogs with functional groups that are not so obviously related to the structure of 4-aminoquinolines also showed substantial improvements in quick-killing potency over azithromycin despite not being able to directly inhibit the chemical polymerization of β-hematin into hemozoin. Additionally, the chloroquinoline-modified analog, GSK-66, maintained moderate inhibitory activity against late-stage gametocytes, whereas chloroquine is poorly inhibitory against these stages (48, 71). Late-stage gametocytes tend to reduce their metabolic activity to housekeeping levels upon reaching maturation. Therefore, it is not completely surprising that chloroquine and drugs with chloroquine-like activity, which rely on active digestion of hemoglobin, lose their efficacy over the course of gametocyte development (48, 71, 81). However, to maintain modest late-stage gametocytocidal activity, GSK-66 would likely need to target additional non-chloroquine-related pathways.

We previously interrogated the mechanism of action of GSK-66 and other azithromycin analogs in trophozoites using metabolomics (22). There, we found broad dysregulation of metabolites associated with not only the parasite’s food vacuole but also the mitochondrion for quick-killing analogs with substituted phenyl and quinoline modifications. Importantly, for the investigation of chloroquine-like activity, metabolomic signatures that are most typical of chloroquine treatment were not prominent in chloroquinoline or non-chloroquinoline azithromycin analog-treated parasites. Although a different set of non-chloroquinoline modified azithromycin analogs were used in the current study, we did not observe any clear patterns in CETSA that would logically cause these metabolomic signatures for this subset of modifications. For the chloroquinoline-containing GSK-66, the metabolomics studies showed that drug treatment caused significant dysregulation of a broad range of metabolic pathways (22). Here, GSK-66 also led to thermal stabilization of the highest number of proteins in the intact-cell CETSA experiments, pointing toward a broad signature of drug-induced killing for this drug, where GSK-66 is likely acting very quickly on several different cellular pathways to disrupt parasite cell homeostasis.

Given the high levels of protein stabilization and broad enrichment of cellular pathways across the intact-cell CETSA experiments, it is not yet possible to link the quick-killing mechanism of action for GSK-66 or the other tested azithromycin analogs to a specific pathway. However, the magnitude of the drug response induced by intact-cell treatment despite the lack of robust lysate hits with the azithromycin analogs, specifically GSK-66, may indicate that the analogs are targeting proteins that require a specific biological environment for binding to be preserved through the intact-cell experiments. Alternatively, these compounds may not directly bind proteins, but instead, disrupt other intracellular processes, leading to widespread modification of protein stability within the parasite. Supporting this, a recent study using an analog identical to GSK-66 reported a high level of drug retention within infected, but not uninfected, human RBCs (29). Hence, it is plausible that these analogs have lipid membrane binding potential, which could impact broadly across parasite cell homeostasis. Another explanation for these results could be that the changes in protein stabilization are impacted by protein abundance changes as a response to treatment. This remains a possibility as we also reported rapid and significant changes in parasite metabolic signatures after treatment with GSK-66 (22).

Considering the urgent need for new anti-malarials, azithromycin analogs may present several favorable characteristics for repurposing. A major advantage is that they can be modified to be fast-acting compounds for treatment of acute cases while maintaining slow-acting properties for chemoprotection (22). Although we did not test for apicoplast targeting delayed-death activity across the five azithromycin analogs tested in this study, we and others have previously shown that the position of the added functional group on GSK-66 removes bacterial ribosome binding, while the positioning of the functional group on UAW-1 to 4 generally maintains apicoplast targeting delayed-death activity (22, 24, 29). Azithromycin analogs with chloroquinoline modifications currently demonstrate the greatest potential for clinical translation. For example, GSK-66 has broad-acting potential with inhibitory activity against mature gametocytes and gamete formation, exceeding the potency of many existing anti-malarials (74). Another study also demonstrates that a structurally identical compound to GSK-66 (known as compound 2 in [29]) possesses favorable *in vivo* efficacy, with low levels of blood clearance, a long half-life, and moderate oral bioavailability (29). Furthermore, we have been unable to select for parasites that have significant resistance *in vitro* to GSK-66 and other quick-killing azithromycin analogs (22). This supports that these azithromycin analogs likely work through multiple modes of action and hence may maintain useful activity even against multidrug-resistant parasites in the field. It is also promising to note that chloroquine-resistant parasites *in vitro* tend to exhibit only modest improvements in survival against chloroquinoline-containing analogs. Additionally, there remains the flexibility to bypass this chloroquine resistance while still maintaining significant improvements in quick-killing potency over azithromycin, by instead incorporating non-chloroquinoline functional groups such as naphthalene. However, further optimization would be needed to improve the potency of non-chloroquinoline drugs down to the level of lead chloroquinoline analogs and also to determine whether they conserve promising activity against late-stage gametocytes, which was not tested here.

In this study, we have provided insights into the quick-killing mechanism of action of azithromycin analogs. Our current findings demonstrate that both chloroquinoline and non-chloroquinoline-containing analogs exhibit inhibitory activity resembling chloroquine’s inhibition of hemoglobin digestion and heme polymerization in chemical assays. Further investigation into their mechanism of action using thermal shift proteomics, however, supports that the mechanism for quick-killing is likely to encompass a much broader range of potential targets beyond that of chloroquine. This broad mechanism of quick-killing likely underpins their ability to maintain potent activity in multidrug-resistant parasites and their resilience to resistance selection *in vitro*. Additionally, this study highlights that chemical modification of azithromycin has the potential to greatly improve gametocytocidal activity, with GSK-66 maintaining moderate inhibitory activity against the relatively drug-resistant late-stage gametocytes. Therefore, this class of azithromycin analogs holds promising potential for repurposing as a fast and broad-acting anti-malarial.

## Data Availability

The mass spectrometry proteomics data have been deposited to the ProteomeXchange Consortium via the PRIDE (82) partner repository with the data set identifier PXD055102.

## SUPPLEMENTAL MATERIAL

File S1 aac.01783-24-s0001.pdf Chemistry methods.

Supplemental figures aac.01783-24-s0002.pdf Figures S1 to S9.

ASM does not own the copyrights to Supplemental Material that may be linked to, or accessed through, an article. The authors have granted ASM a non-exclusive, world-wide license to publish the Supplemental Material files. Please contact the corresponding author directly for reuse.
