# Supplementary material for: Characterizing the quick-killing mechanism of action of azithromycin analogs against malaria parasites
Source: Antimicrob Agents Chemother. 2025 Jul 25;69(9):e01783-24. doi: 10.1128/aac.01783-24 (PMC12406678; doi:10.1128/aac.01783-24)
Supplement: File S1 — Chemistry methods. [file aac.01783-24-s0001.pdf]

Chemistry Experimental

General Chemistry Methods. NMR spectra were recorded on a Bruker Ascend™ 300. Chemical shifts are reported in ppm on the δ scale and referenced to the appropriate solvent peak. DMSO-$d_6$and $CDCl_3$ contain H_2O. Chromatography was performed with silica gel 60 (particle size 0.040-0.063 µm) using an automated CombiFlash Rf Purification System. LCMS were recorded on an Agilent LCMS system comprised of an Agilent G6120B Mass Detector, 1260 Infinity G1312B Binary pump, 1260 Infinity G1367E HiPALS autosampler and 1260 Infinity G4212B Diode Array Detector. Conditions for LCMS were as follows, column: Luna® Omega 3 µm PS C18 100 Å, LC Column 50 × 2.1 mm at 20 ºC, injection volume 2 µL, gradient: 5-100% B over 3 min (solvent A: H_2O 0.1% formic acid; solvent B: ACN 0.1% formic acid), flow rate: 1.5 mL/min, detection: 254 nm, acquisition time: 4.3 min. **UAW-1** and **UAW-2** were found to be >97% pure by this method. **UAW-3** and **UAW-4** (not UV active) were found to be >90% pure by NMR acquired as described above.

Chemistry Procedures.

Int-1

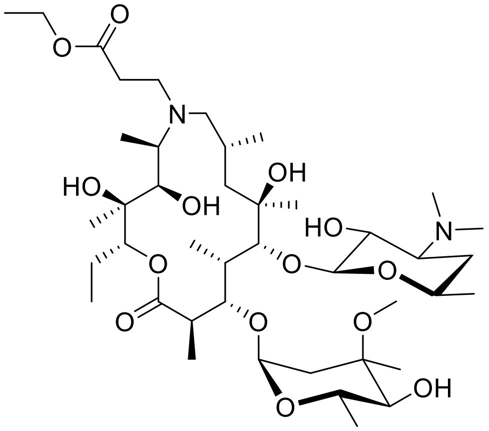

9-Deoxo-9-dihydro-9a-(N'-ethylproprionate)-9a-aza-9a-homoerythromycin A (**Int-1**). Ethyl acrylate (18.1 mL, 170 mmol) was added to a solution of 9-deoxo-9a-aza-9a-homoerythromycin A (2.50 g, 3.40 mmol) in $CHCl_3$ (50 mL). Reaction mixture was stirred at 60 °C for 48 h. The reaction mixture was then concentrated, and the crude purified by column chromatography eluting

with 100% DCM to DCM: MeOH: NH_4OH (90: 5: 0.5) to afford **Int-1** as a solid (1.65 g, 58%). ^1H NMR (300 MHz, $CDCl_3$): δ 4.96 (d, *J* 4.5 Hz, 1H), 4.81 (dd, *J* 2.2, 10.10 Hz, 1H), 4.46 (d, *J* 7.3 Hz, 1H), 3.99 - 4.25 (m, 5H), 3.70 - 3.79 (m, 1H), 3.65 (d, *J* 6.6 Hz, 1H), 3.35 - 3.58 (m, 3H), 3.33 (s, 3H), 3.26 (dd, *J* 7.3, 10.2 Hz, 1H), 3.03 (t, *J* 9.8 Hz, 1H), 2.74 - 2.91 (m, 2H), 2.55 - 2.72 (m, 4H), 2.41 - 2.55 (m, 2H), 2.31 (s, 6H), 2.15 - 2.27 (m, 2H), 1.95 - 2.09 (m, 2H), 1.81 - 1.95 (m, 2H), 1.54 - 1.76 (m, 5H), 1.35 - 1.53 (m, 2H), 1.32 (app t, *J* 4.6 Hz, 6H), 1.20 - 1.26 (m, 10H), 1.05 - 1.18 (m, 9H), 0.84 - 1.00 (m, 6H). LCMS *m/z* 836.5 [M+1].

Int-2

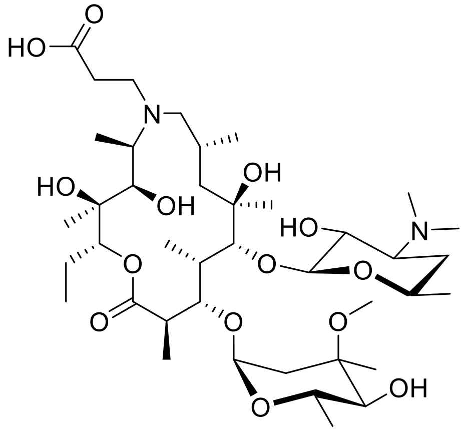

9-Deoxo-9-dihydro-9a-(N'-propionic acid)-9a-aza-9a-homoerythromycin A (**Int-2**). A solution of lithium hydroxide (109 mg, 4.54 mmol) in H_2O (25 mL) was added to a solution of **Int-1** (1.65 g, 1.98 mmol) in THF (25 mL). The reaction mixture was stirred at 20 °C for 2 h. THF was then removed in vacuo and brine was added to the reaction mixture (30 mL). This mixture was then extracted with DCM (3 × 30 mL). The combined organic extracts were dried over anhydrous Na_2SO_4, filtered and concentrated to afford **Int-2** as a solid (1.59 g, 100%). ^1H NMR (300 MHz, DMSO-d_6): δ 5.04 (dd, *J* 2.0, 11.0 Hz, 1H), 4.85 (d, *J* 4.67 Hz, 1H), 4.38 (d, *J* 7.3 Hz, 1H), 4.25 (d, *J* 7.7 Hz, 1H), 4.07 - 4.11 (m, 1H), 4.05 (s, 1H), 4.01 (d, *J* 7.1 Hz, 1H), 3.70 (s, 1H), 3.56 - 3.67 (m, 1H), 3.48 - 3.55 (m, 2H), 3.45 (br s, 1H), 3.22 (s, 3H), 2.96 - 3.06 (m, 1H), 2.90 (t, *J* 8.5 Hz, 1H), 2.70 - 2.82 (m, 1H), 2.24 - 2.45 (m, 4H), 2.21 (s, 6H), 2.03 - 2.19 (m, 2H), 1.87 - 1.99 (m, 2H), 1.71 - 1.87 (m, 2H), 1.50 - 1.61 (m, 2H), 1.40 - 1.50 (m, 2H), 1.23 - 1.39 (m, 2H), 1.05 - 1.22

(m, 17H), 0.98 (d, *J* 6.7 Hz, 3H), 1.01 (d, *J* 7.63 Hz, 3H), 0.91 (s, 3H), 0.72 - 0.82 (m, 6H). LCMS *m/z* 807.4 [M+1].

Int-3

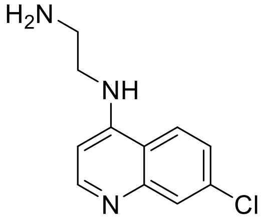

N'-(7-chloro-4-quinolyl)ethane-1,2-diamine (**Int-3**). Ethylenediamine (0.843 mL, 12.6 mmol) was dissolved in 4,7-dichloroquinoline (500 mg, 2.52 mmol) and stirred at 110 °C for 2 h. The reaction was then cooled to 20 °C and then concentrated. The crude mixture was then dissolved in EtOAc (40 mL) and washed with 10% $NaHCO_3$ (15 mL), brine (15 mL), dried with anhydrous Na_2SO_4, filtered and then concentrated to obtain **Int-3** as an oil which solidified (510 mg, 91%). ^{1}H NMR (300 MHz, $CDCl_3$): δ 8.55 (d, *J* 5.4 Hz, 1H), 7.97 (d, *J* 2.1 Hz, 1H), 7.74 (d, *J* 8.9 Hz, 1H), 7.38 (dd, *J* 2.2, 8.9 Hz, 1H), 6.43 (d, *J* 5.4 Hz, 1H), 5.76 (br s, 1H), 3.28 - 3.41 (m, 2H), 3.08 - 3.20 (m, 2H). LCMS *m/z* 222.2 [M+1].

UAW-1

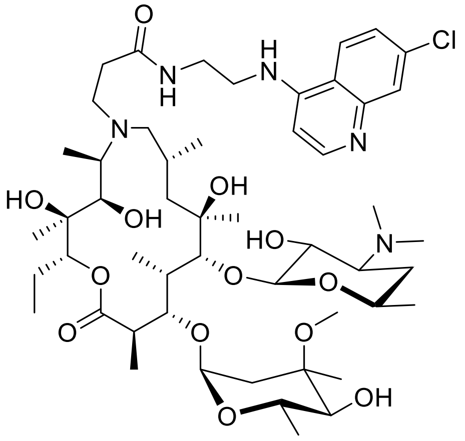

9-Deoxo-9-dihydro-9a-(N'(N-(2-((7-chloroquinolin-4-yl)amino)ethyl)propionamide))-9a-aza-9a-homoerythromycin A (**UAW-1**). **Int-2** (100 mg, 0.124 mmol), HATU (59 mg, 0.16 mmol), **Int-3** (23.0 mg, 0.104 mmol) and DIPEA (36 µL, 0.21 mmol) were dissolved in DMF (2 mL) and stirred for 2 h. The reaction was then quenched with 10% citric acid (1 mL), concentrated and dissolved in EtOAc (30 ml). The organics was washed with $NaHCO_3$ (20 mL), brine (20 mL), dried with Na_2SO_4 and concentrated. The crude was purified by column chromatography eluting with 100% DCM to 23% MeOH (with 10% NH_4OH) to obtain **UAW-1** as a solid (47 mg, 44%). ^1H NMR (300 MHz, $CDCl_3$): δ 8.47 (d, *J* 5.6 Hz, 1H), 7.94 (d, *J* 2.1 Hz, 1H), 7.86 (d, *J* 9.0 Hz, 1H), 7.67 (br s, 1H), 7.37 (dd, *J* 2.1, 8.9 Hz, 1H), 7.09 (br. s., 1H), 6.35 (d, *J* 5.6 Hz, 1H), 4.91 (d, *J* 4.6 Hz, 1H), 4.63 (d, *J* 9.1 Hz, 1H), 4.40 - 4.49 (m, 1H), 4.24 (d, *J* 6.6 Hz, 1H), 3.98 - 4.16 (m, 2H), 3.84 (d, *J* 8.7 Hz, 1H), 3.59 - 3.72 (m, 4H), 3.47 - 3.57 (m, 2H), 3.30 - 3.45 (m, 5H), 3.20 - 3.26 (m, 1H), 3.04 (d, *J* 9.2 Hz, 1H), 2.70 - 2.97 (m, 4H), 2.34 - 2.57 (m, 6H), 2.30 (s, 6H), 1.95 - 2.09 (m, 2H), 1.76 - 1.93 (m, 2H), 1.62 - 1.76 (m, 2H), 1.44 - 1.59 (m, 2H), 1.31 - 1.38 (m, 6H), 1.15 - 1.28 (m, 11H), 1.06 - 1.14 (m, 6H), 0.91 - 1.06 (m, 6H), 0.82 - 0.89 (m, 3H). LCMS *m/z* 1010.4 [M+1]. HRMS acquired: (M + H) 1010.5795; $C_{51}H_{84}ClN5O_{13}$ requires (M + H), 1010.5827.

UAW-2

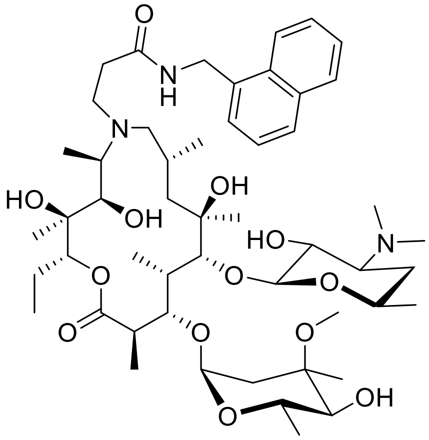

9-Deoxo-9-dihydro-9a-(N'(N-(naphthalen-1-ylmethyl)propionamide))-9a-aza-9a-homoerythromycin A (**UAW-2**). The procedure used for **UAW-1** was followed using 1-naphthylmethylamine (58 mg, 0.055 mmol) and **Int-2** (100 mg, 0.124 mmol) to afford **UAW-2** as a solid (45 mg, 38%). ^1H NMR (300 MHz, $CDCl_3$): δ 8.10 (d, *J* 8.17 Hz, 1H), 7.82 - 7.90 (m, 1H),

7.79 (d, *J* 8.1 Hz, 1H), 7.32 - 7.65 (m, 4H), 6.71 (br s, 1H), 5.20 (dd, *J* 6.8, 14.8 Hz, 1H), 4.68 - 4.95 (m, 3H), 4.58 (br. s., 1H), 4.41 (d, *J* 7.3 Hz, 1H), 4.15 (dd, *J* 2.2, 7.6 Hz, 1H), 4.04 (dd, *J* 6.3, 9.2 Hz, 1H), 3.69 (br s, 2H), 3.60 (d, *J* 6.0 Hz, 1H), 3.37 - 3.48 (m, 2H), 3.10 - 3.37 (m, 5H), 2.72 - 2.95 (m, 5H), 2.28 - 2.64 (m, 12H), 2.20 (d, *J* 9.87 Hz, 1H), 1.84 - 2.06 (m, 3H), 1.66 (d, *J* 13.3 Hz, 2H), 1.38 - 1.55 (m, 3H), 1.18 - 1.31 (m, 16H), 1.05 - 1.18 (m, 9H), 0.99 (d, *J* 7.0 Hz, 3H), 0.90 (t, *J* 7.3 Hz, 3H). LCMS *m/z* 946.6 [M+1]. HRMS acquired: (M + H) 946.5989; $C_{51}H_{83}N_3O_{13}$ requires (M + H), 946.5999.

UAW-3

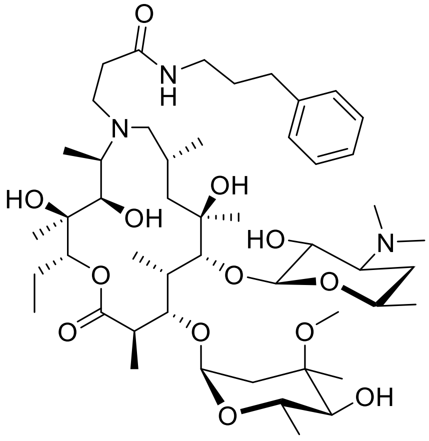

9-Deoxo-9-dihydro-9a-(N'(N-(3-phenylpropyl)propionamide))-9a-aza-9a-homoerythromycin A (**UAW-3**). The procedure used for **UAW-1** was followed using 3-phenylpropylamine (23 mg, 0.169 mmol) and **Int-2** (150 mg, 0.186 mmol) to afford **UAW-3** as a solid (49 mg, 31%). ^{1}H NMR (300 MHz, $CDCl_3$): δ 7.12 - 7.30 (m, 5H), 4.75 - 4.96 (m, 2H), 4.49 (d, *J* 7.1 Hz, 1H), 3.96 - 4.15 (m, 2H), 3.63 (dd, *J* 14.5, 19.9 Hz, 4H), 3.22 - 3.41 (m, 6H), 2.97 - 3.11 (m, 2H), 2.80 - 2.93 (m, 2H), 2.66 (t, *J* 7.8 Hz, 4H), 2.45 (br s, 6H), 2.34 (d, *J* 15.2 Hz, 3H), 2.17 - 2.26 (m, 2H), 1.97 - 2.13 (m, 2H), 1.65 – 1.97 (m, 6H), 1.43 - 1.64 (m, 3H), 1.17 - 1.42 (m, 20H), 0.94 - 1.15 (m, 11H), 0.85 - 0.92 (m, 3H). LCMS *m/z* 924.6 [M+1]. HRMS acquired: (M + H) 924.6128; $C_{49}H_{85}N_3O_{13}$ requires (M + H), 924.6155.

UAW-4

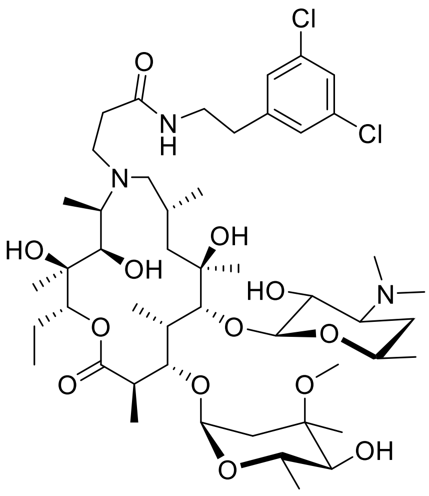

9-Deoxo-9-dihydro-9a-(N'(N-(3,5-dichlorophenethyl)propionamide))-9a-aza-9a-homoerythromycin A **(UAW-4)**. The procedure used for **UAW-1** was followed using 2-(3,5-dichlorophenyl)ethanamine (32 mg, 0.169 mmol) and **Int-2** (150 mg, 0.186 mmol) to afford **UAW-4** as a solid (48 mg, 29%). ^{1}H NMR (300 MHz, $CDCl_3$): δ 7.15 - 7.25 (m, 3H), 4.72 - 4.97 (m, 2H), 4.50 (d, *J* 7.3 Hz, 1H), 3.95 - 4.17 (m, 2H), 3.51 - 3.73 (m, 5H), 3.41 - 3.51 (m, 3H), 3.25 - 3.39 (m, 4H), 3.07 (t, *J* 9.5 Hz, 1H), 2.64 - 2.92 (m, 6H), 2.45 (br s, 6H), 2.32 (d, *J* 14.5 Hz, 3H), 2.14 - 2.26 (m, 2H), 1.82 - 2.14 (m, 6H), 1.76 (d, *J* 13.6 Hz, 1H), 1.41 - 1.65 (m, 3H), 1.29 - 1.41 (m, 8H), 1.20 - 1.27 (m, 8H), 1.11 - 1.19 (m, 5H), 1.00 - 1.11 (m, 9H), 0.88 (t, *J* 7.3 Hz, 3H). LCMS *m/z* 978.4 [M+1], 980.6. HRMS acquired: (M + H) 978.5196; $C_{48}H_{81}Cl_2N_3O_{13}$ requires (M + H), 978.5219.

Synthesis of GSK-66

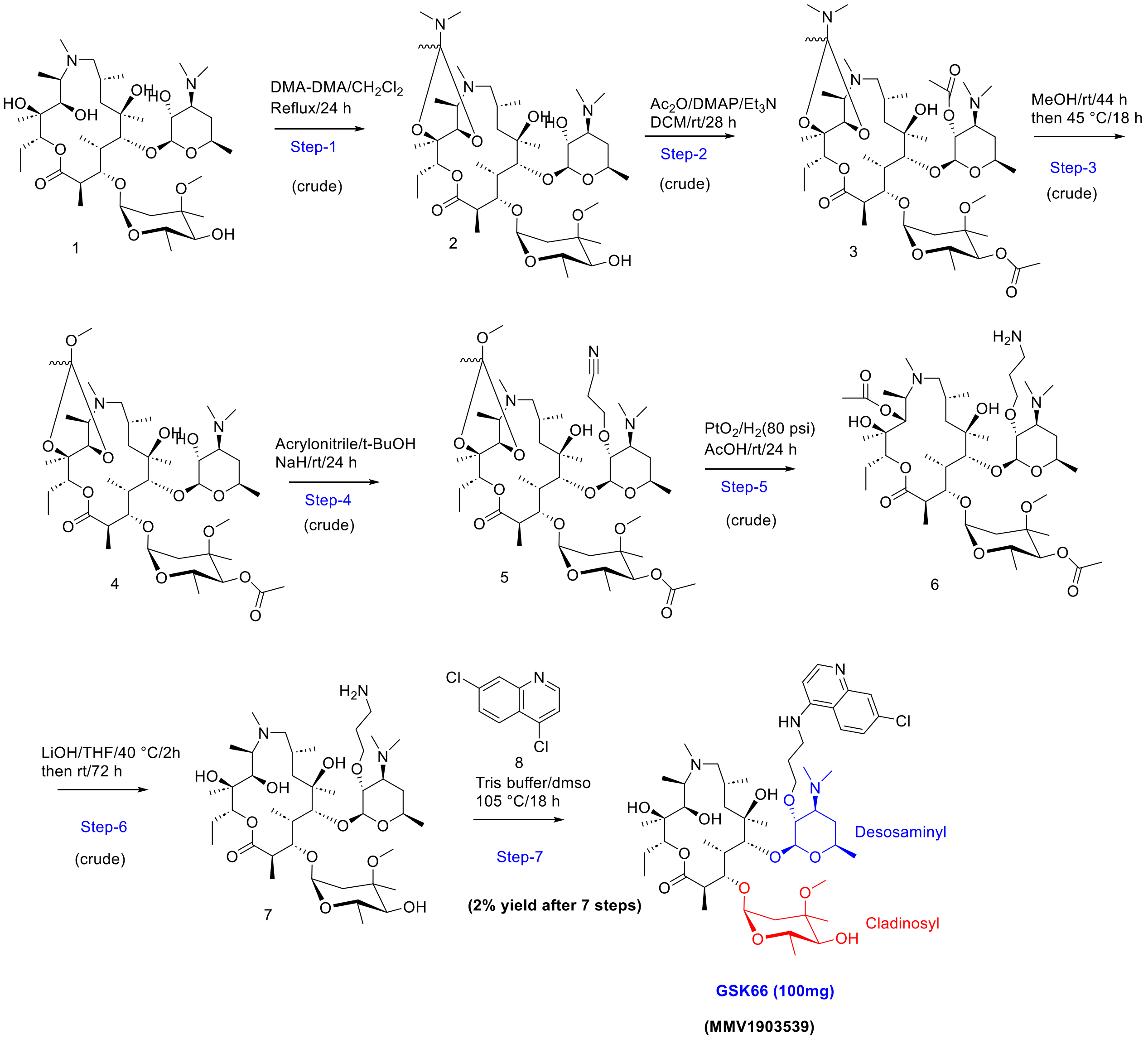

Step-1: ***Synthesis of 11,12-O-[1-(Dimethylamino)ethylidene]-9-deoxo-9a-methyl-9a-aza-9ahomoerythromycin*** **(2)**

9-Deoxo-9a-methyl-9a-aza-9a-homoerythromycin (**1**) (4 g, 5.34 mmol) was dissolved in dry DCM (25 mL). The DMA/DMA (3.70 g, 27.79 mmol) was added in one portion, and then heated at reflux temperature for 24 h under argon. The solvent was evaporated to afford title product 11,12-*O*-[1-(dimethylamino)ethylidene]-9-deoxo-9a-methyl-9a-aza-9a- homoerythromycin (**2**) (4.2 g crude) as light brown gummy liquid which was used for the next step without further purification. LC-MS: *m/z* =818.30 $[M+H]^+$.

Step-2: ***Synthesis of compound 2',4''-di-O-Acetyl-11,12-O-[1-(dimethylamino)ethylidene]-9-deoxo-9a-methyl-9a-aza-9ahomoerythromycin*** **(3)**

To a stirred solution of crude 11,12-*O*-[1-(dimethylamino)ethylidene]-9-deoxo-9a-methyl-9a-aza-9ahomoerythromycin (**2**) (4 g, 4.89 mmol) in dry DCM (25 mL) were successively added Et_3N (2.39 mL, 17.12 mmol), DMAP (0.24 g, 1.96 mmol) and Ac_2O (1.16 mL, 12.23 mmol) at 0 °C and the reaction mixture was allowed to slowly reach rt and the reaction mixture was stirred at rt for 28 h. The reaction mixture was washed with H_2O (80 mL). DCM layers were dried over Na_2SO_4 and concentrated under vacuum and the resultant crude residue was washed with diethyl ether to obtain crude 2',4''-di-*O*-acetyl-11,12-*O*-[1-(dimethylamino)ethylidene]-9-deoxo-9a-methyl-9a-aza-9a-homoerythromycin (**3**) (3.1 g, crude) as off white gummy solid which was used for the next step as such. LC-MS: *m/z* =902.40 $[M+H]^+$.

Step-3: ***Synthesis of compound 4''-O-Acetyl-11,12-O-(methoxyethylidene)-9-deoxo-9a-methyl-9a-aza-9ahomoerythromycin*** **(4)**

The crude compound 2',4''-di-*O*-acetyl-11,12-*O*-[1-(dimethylamino)ethylidene]-9-deoxo-9a-methyl-9a-aza-9ahomoerythromycin (**3**) (3.0 g, 3.33 mmol) was dissolved in MeOH (75 mL) under argon and stirred at rt for 44 h, then at 45 °C for 18 h. MeOH was evaporated under vacuum and the crude product was precipitated from Et_2O-hexane to afford crude 4''-*O*-acetyl-11,12-*O*-(methoxyethylidene)-9-deoxo-9a-methyl-9a-aza-9a-homoerythromycin (**4**) (2.5 g, crude) as white solid which was used for the next step- as such. LC-MS: *m/z* =847.70 $[M+H]^+$.

Step-4: ***Synthesis of 4''-O-Acetyl-11,12-O-(methoxyethylidene)-2'-O-cyanoethyl-9-deoxo-9a-methyl-9a-aza-9a-homoerythromycin (5)***

To a solution of crude 4''-*O*-acetyl-11,12-O-(methoxyethylidene)-9-deoxo-9a-methyl-9a-aza-9a-homoerythromycin (**4**) (1.8 g, 2.12 mmol) in acrylonitrile (11.15 mL, 170.2 mmol), *t*-BuOH (1 mL) was added at rt under nitrogen and the reaction mixture was cooled to 0 °C. NaH (60% in mineral oil, 0.12 g) was added in small amounts during 0.5 h. The temperature was allowed to slowly reach to rt. After 24 h of stirring at rt under argon, excess acrylonitrile was evaporated under reduced pressure. The polymer of acrylonitrile was precipitated in EtOAc/n-hexane and

filtered off. The mother liquor was evaporated to afford oily product, which was then dissolved in EtOAc and extracted with water. The combined organic layers washed with water, brine, dried over Na_2SO_4, filtered, and concentrated to get crude product, which was dissolved in minimum amount of DCM, then Hexane (30 mL) was added. The suspension was filtered over Celite bed, washed with DCM. The solvent was evaporated under vacuum to afford 4"-*O*-acetyl-11,12-*O*-(methoxyethylidene)-2'-*O*-cyanoethyl-9-deoxo-9a-methyl-9a-aza-9a-homoerythromycin (**5**) (1.8 g crude) as light brown gummy liquid which was used for the next step as such. LC-MS: *m/z* =900.10 $[M+H]^+$.

Step-5: ***Synthesis of 4",11-Di-O-acetyl-2'-O-(3-aminopropyl)-9-deoxo-9a-methyl-9a-aza-9a-homoerythromycin (6)***

The crude compound 4"-*O*-acetyl-11,12-*O*-(methoxyethylidene)-2'-*O*-cyanoethyl-9-deoxo-9a-methyl-9a-aza-9a-homoerythromycin (**5**) crude) (1.8 g, 2.0 mmol) was dissolved in glacial HOAc (30 mL) and hydrogenated with PtO_2 (0.3 g) under H_2 atmosphere (80 psi) at rt. After 24 h, the catalyst was filtered off and mother liquor evaporated under reduced pressure. The residue was dissolved in water and DCM (100 mL, 1:1 mixture), the layers were separated, and pH of H_2O layer was adjusted to 6.3 and washed with DCM (2 × 50 mL) than pH of H2O solution was increased to 8.6 and extracted with DCM (2 × 50 mL). The combined organic layer was washed with brine, dried over Na_2SO_4 and concentrated to afford 4",11-di-*O*-acetyl-2'-*O*-(3-aminopropyl)-9-deoxo-9a-methyl-9a-aza-9ahomoerythromycin (**6**) (1.5 g, crude) as a off white solid which was used of the next step as such. LC-MS: *m/z* =890.50 $[M+H]^+$.

Step-6: ***Synthesis of 2'-O-(3-Aminopropyl)-9-deoxo-9a-methyl-9a-aza-9a-homoerythromycin (7)***

The crude compound 4",11-di-*O*-acetyl-2'-*O*-(3-aminopropyl)-9-deoxo-9a-methyl-9a-aza-9ahomoerythromycin (**6**) (1 g, 1.12 mmol) was dissolved in THF/H_2O (1:1, 20 mL), and $LiOH.H_2O$ (0.27 g, 11.23 mmol) was added at rt, heated at 40 °C for 2 h, and stirred at rt for an additional 72 h. H_2O (10 mL) was added to the reaction mixture, followed by extraction with EtOAc. Organic layers were collected, washed with brine, dried over Na_2SO_4, filtered and concentrated to afford 2′-*O*-(3-aminopropyl)-9-deoxo-9a-methyl-9a-aza-9a-homoerythromycin

(**7**) (800 mg as crude) as off white solid which was sued for the next step as such.LC-MS: *m/z* =806.60 $[M+H]^+$.

Step-7: ***Synthesis of 2′-O-{3-[(7-Chloro-4-quinolinyl)amino]propyl}-9-deoxo-9a-methyl-9a-aza-9a-homoerythromycin (GSK66).***

To a solution of 2′-*O*-(3-aminopropyl)-9-deoxo-9a-methyl-9a-aza-9a-homoerythromycin (**7**) (1 g, 1.23 mmol) in DMSO (8 mL) were added 4,7-dichloroquinoline (1.22 g, 6.19 mmol) and Trisma Base (750 mg, 6.19 mmol). The reaction mixture was heated at 105 °C for 18 h. The reaction mixture was cooled to rt and the crude reaction mixture was directly purified via preparative HPLC to afford 2′-*O*-{3-[(7-chloro-4-quinolinyl)amino]propyl}-9-deoxo-9a-methyl-9a-aza-9a-homoerythromycin (60 mg, 2% yield for 7 steps) as white solid. LC-MS: *m/z* =967.56 $[M+H]^+$.

^{1}H NMR, LCMS and HRMS spectra

UAW-1

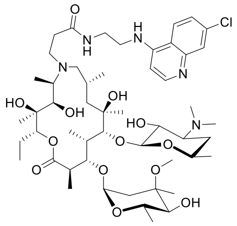

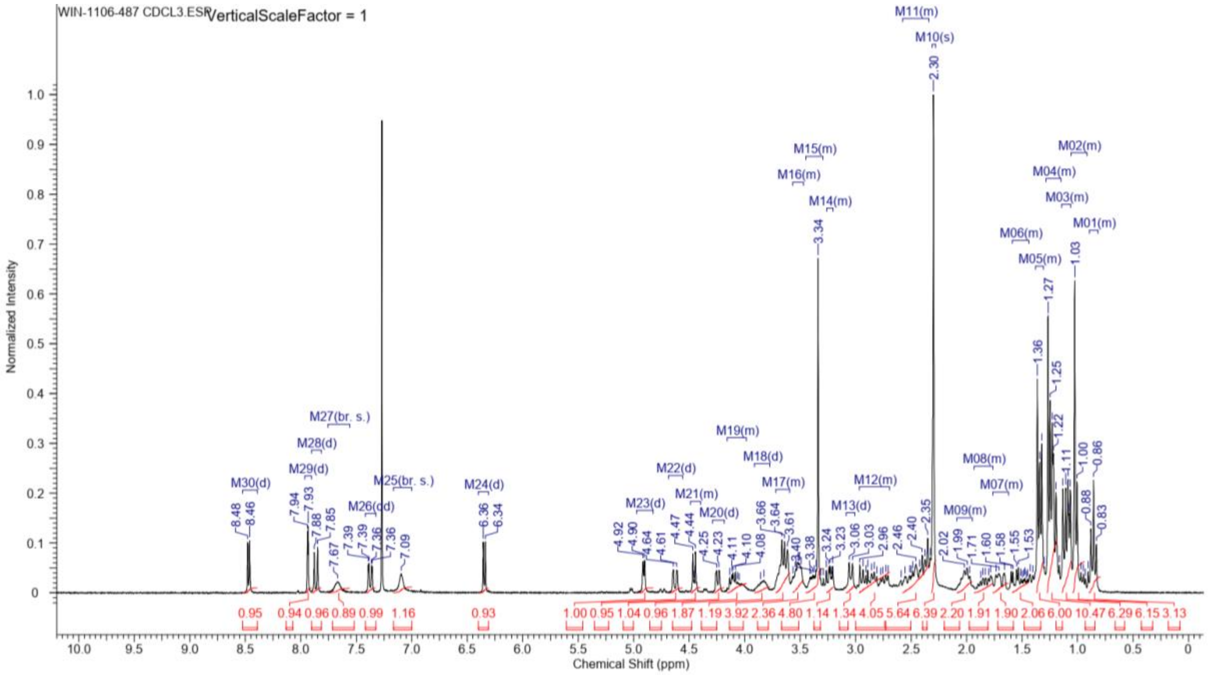

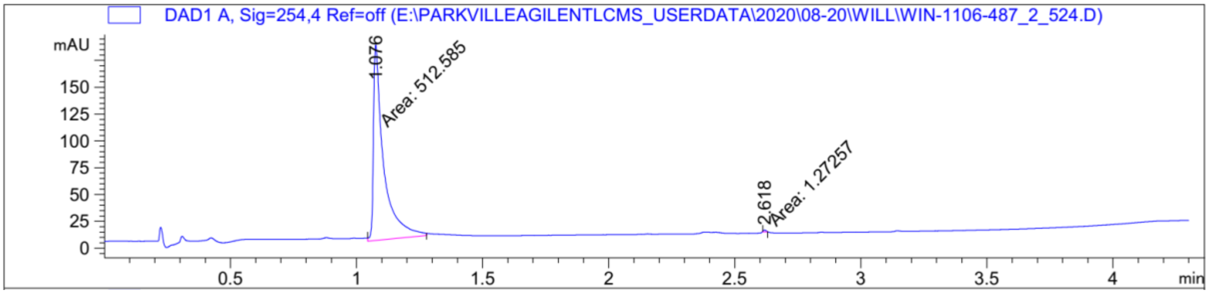

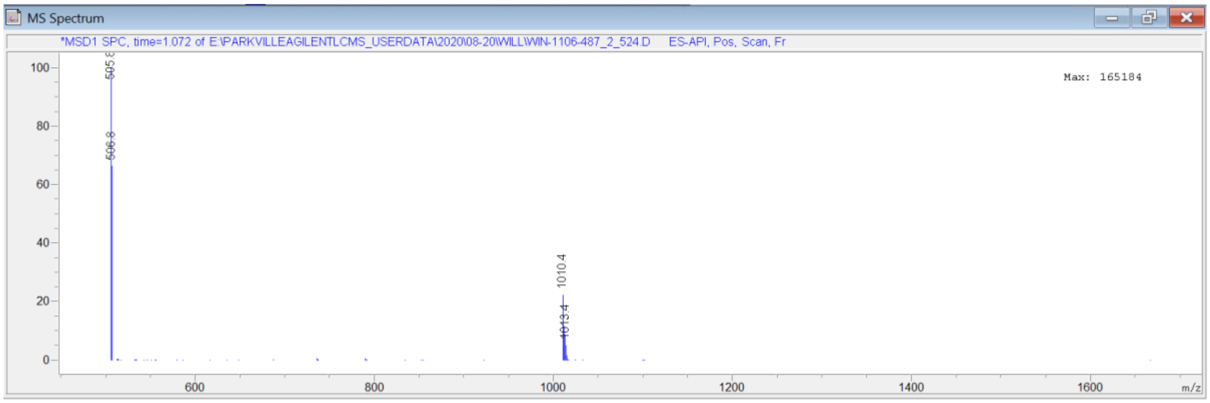
MS Spectrum
*MSD1 SPC, time=1.072 of E:\PARKVILLEAGILENTLCMS_USERDATA\2020\08-20\WILL\WIN-1106-487_2_524.D ES-API, Pos, Scan, Fr
Max: 165184
1010.4
1013.4
506.8
m/z

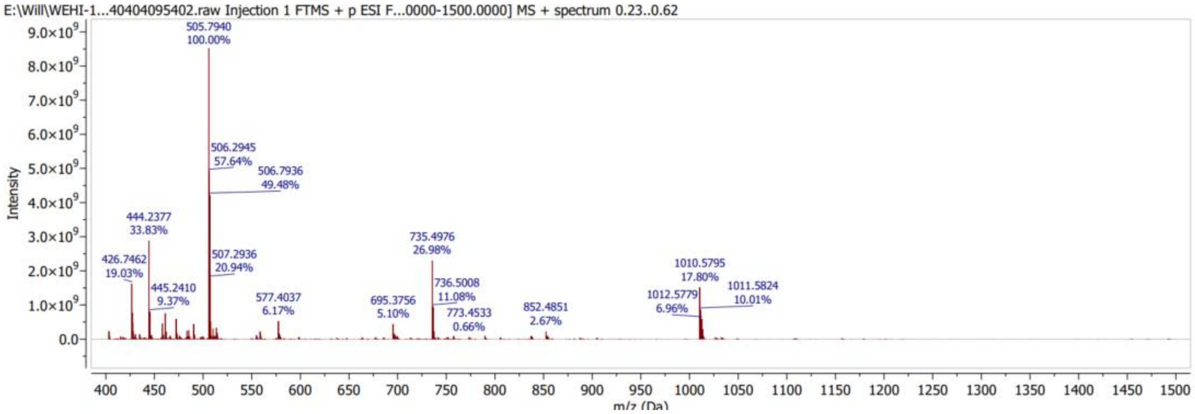
E:\Will\WEHI-1...40404095402.raw Injection 1 FTMS + p ESI F...0000-1500.0000] MS + spectrum 0.23..0.62
Intensity
505.7940
100.00%
506.2945
57.64%
506.7936
49.48%
444.2377
33.83%
426.7462
19.03%
445.2410
9.37%
507.2936
20.94%
577.4037
6.17%
695.3756
5.10%
735.4976
26.98%
736.5008
11.08%
773.4533
0.66%
852.4851
2.67%
1010.5795
17.80%
1012.5779
6.96%
1011.5824
10.01%
m/z (Da)

UAW-2

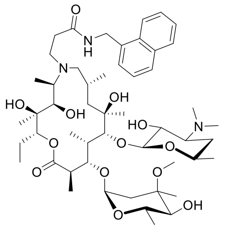
O
N
H
N
HO
OH
OH
HO
N
O
O
O
O
O
O
O
OH

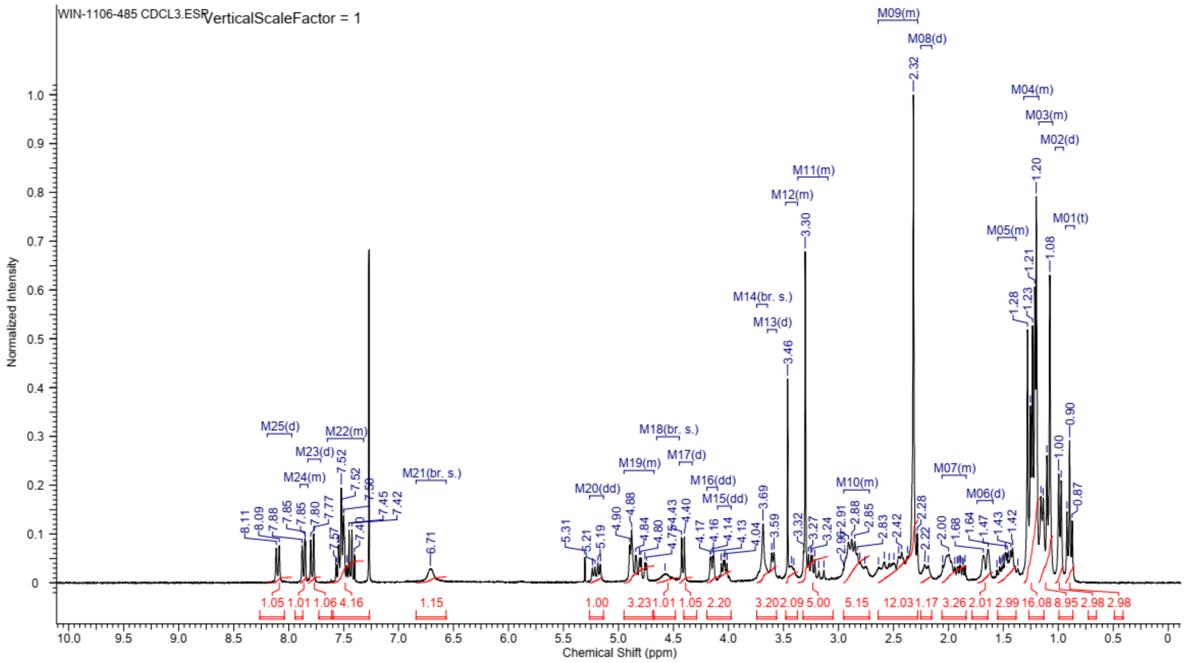
WIN-1106-485 CDCL3.ESP
VerticalScaleFactor = 1
Normalized Intensity
Chemical Shift (ppm)
M01(t)
M02(d)
M03(m)
M04(m)
M05(m)
M06(d)
M07(m)
M08(d)
M09(m)
M10(m)
M11(m)
M12(m)
M13(d)
M14(br. s.)
M15(dd)
M16(dd)
M17(d)
M18(br. s.)
M19(m)
M20(dd)
M21(br. s.)
M22(m)
M23(d)
M24(m)
M25(d)
1.05
1.01
1.06
4.16
1.15
1.00
3.23
1.01
1.05
2.20
3.20
2.09
5.00
5.15
12.03
1.17
3.26
2.01
2.99
16.08
8.95
2.98
2.98

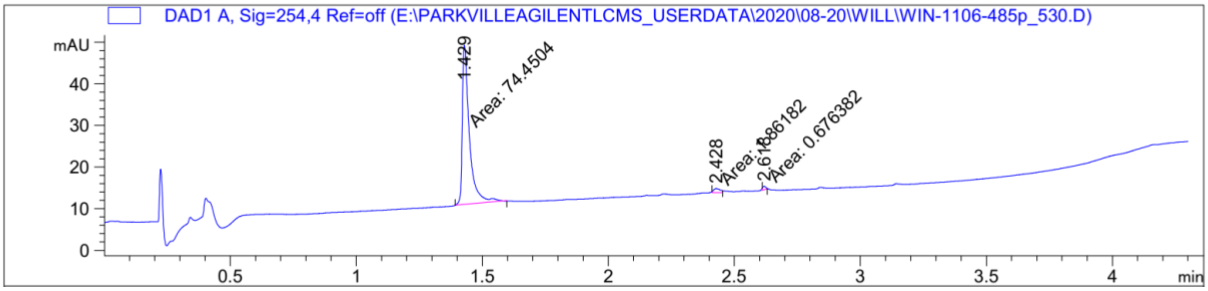
DAD1 A, Sig=254,4 Ref=off (E:\PARKVILLEAGILENTLCMS_USERDATA\2020\08-20\WILL\WIN-1106-485p_530.D)
mAU
1.429
Area: 74.4504
2.428
Area: 0.676382
min

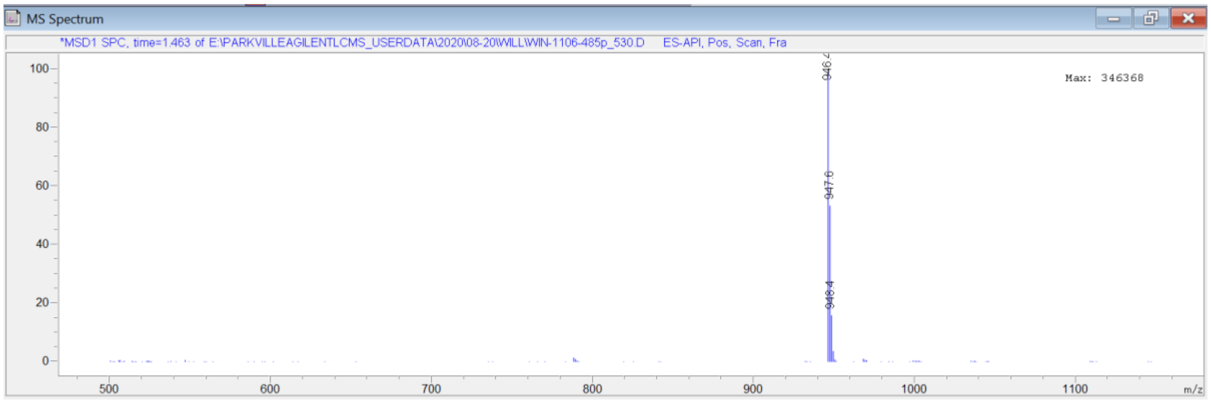
MS Spectrum
*MSD1 SPC, time=1.463 of E:\PARKVILLEAGILENTLCMS_USERDATA\2020\08-20\WILL\WIN-1106-485p_530.D ES-API, Pos, Scan, Fra
Max: 346368
947.6
948.4
m/z

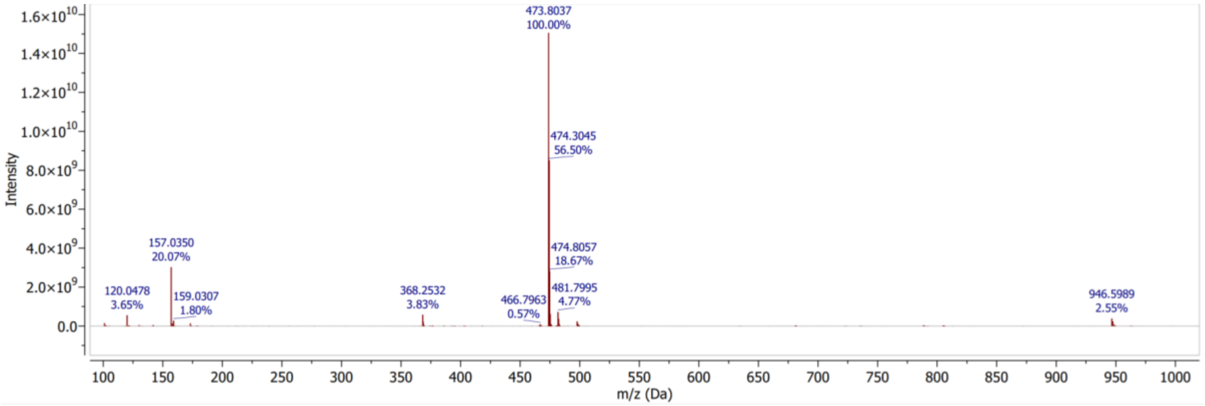
473.8037
100.00%
474.3045
56.50%
474.8057
18.67%
157.0350
20.07%
120.0478
3.65%
159.0307
1.80%
368.2532
3.83%
466.7963
0.57%
481.7995
4.77%
946.5989
2.55%
Intensity
m/z (Da)

UAW-3

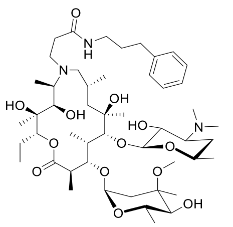
O
N
H
N
HO
OH
OH
HO
N
O
O
O
O
O
O
O
O
OH

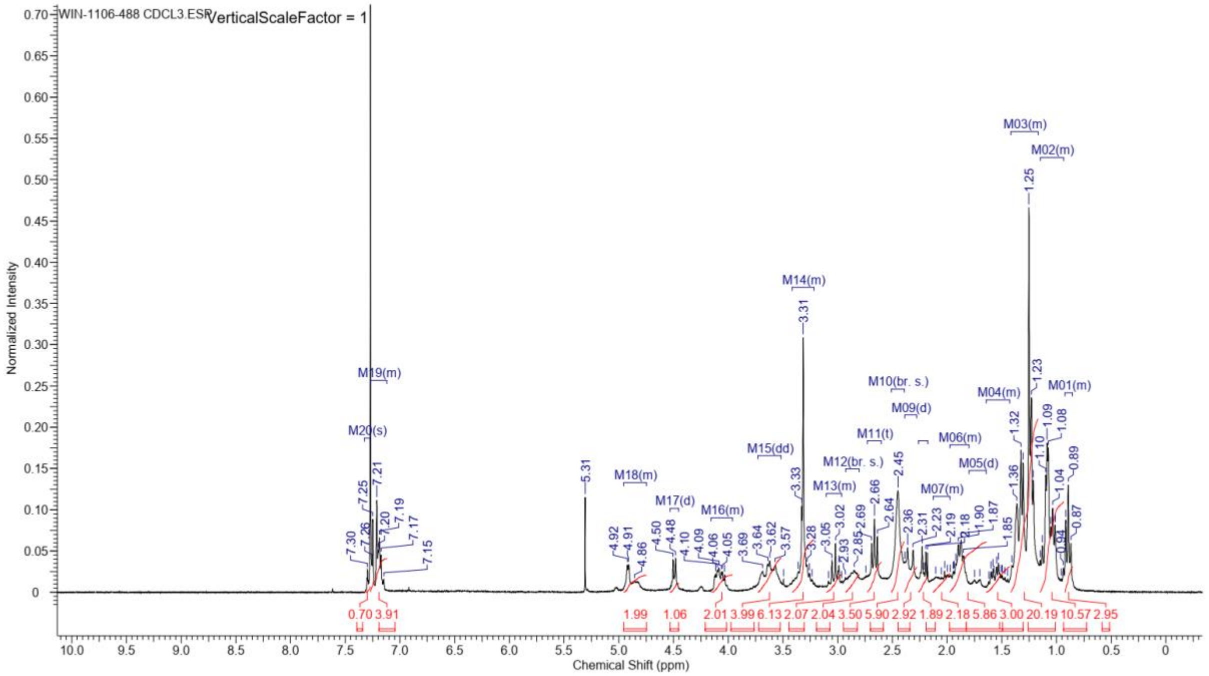
WIN-1106-488 CDCL3.ESP
VerticalScaleFactor = 1
Normalized Intensity
Chemical Shift (ppm)
M01(m)
M02(m)
M03(m)
M04(m)
M05(d)
M06(m)
M07(m)
M09(d)
M10(br. s.)
M11(t)
M12(br. s.)
M13(m)
M14(m)
M15(dd)
M16(m)
M17(d)
M18(m)
M19(m)
M20(s)
0.70 3.91 1.99 1.06 2.01 3.99 6.13 2.07 2.04 3.50 5.90 2.92 1.89 2.18 5.86 3.00 20.19 10.57 2.95

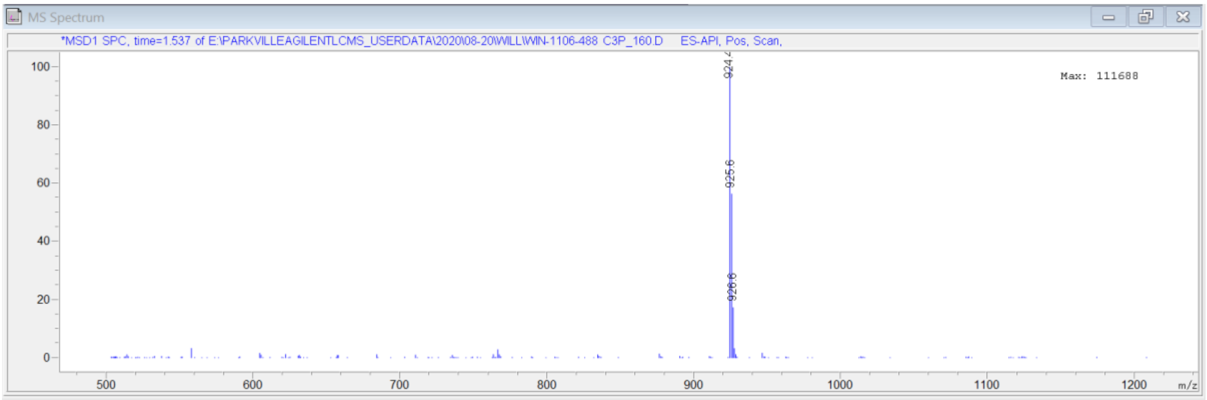
MS Spectrum
*MSD1 SPC, time=1.537 of E:\PARKVILLEAGILENTLCMS_USERDATA\2020\08-20\WILL\WIN-1106-488 C3P_160.D ES-API, Pos, Scan,
Max: 111688
924
925.6
926.6
m/z

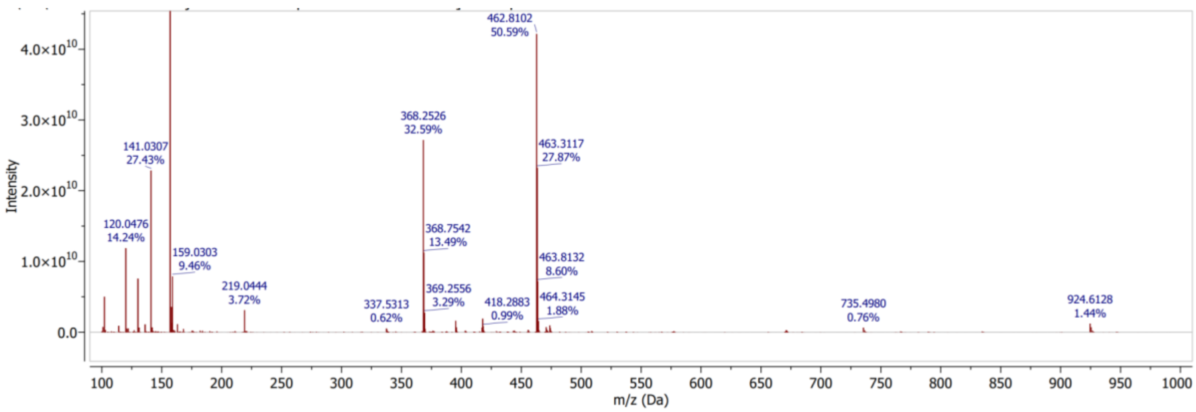
462.8102
50.59%
368.2526
32.59%
141.0307
27.43%
463.3117
27.87%
120.0476
14.24%
368.7542
13.49%
159.0303
9.46%
463.8132
8.60%
219.0444
3.72%
369.2556
3.29%
464.3145
1.88%
337.5313
0.62%
418.2883
0.99%
735.4980
0.76%
924.6128
1.44%
Intensity
m/z (Da)

UAW-4

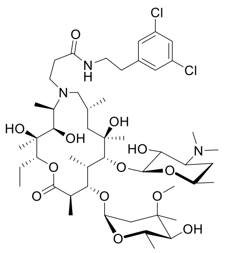
Cl
Cl
O
N
H
N
HO
OH
OH
OH
HO
N
O
O
O
O
O
O
O
O
OH

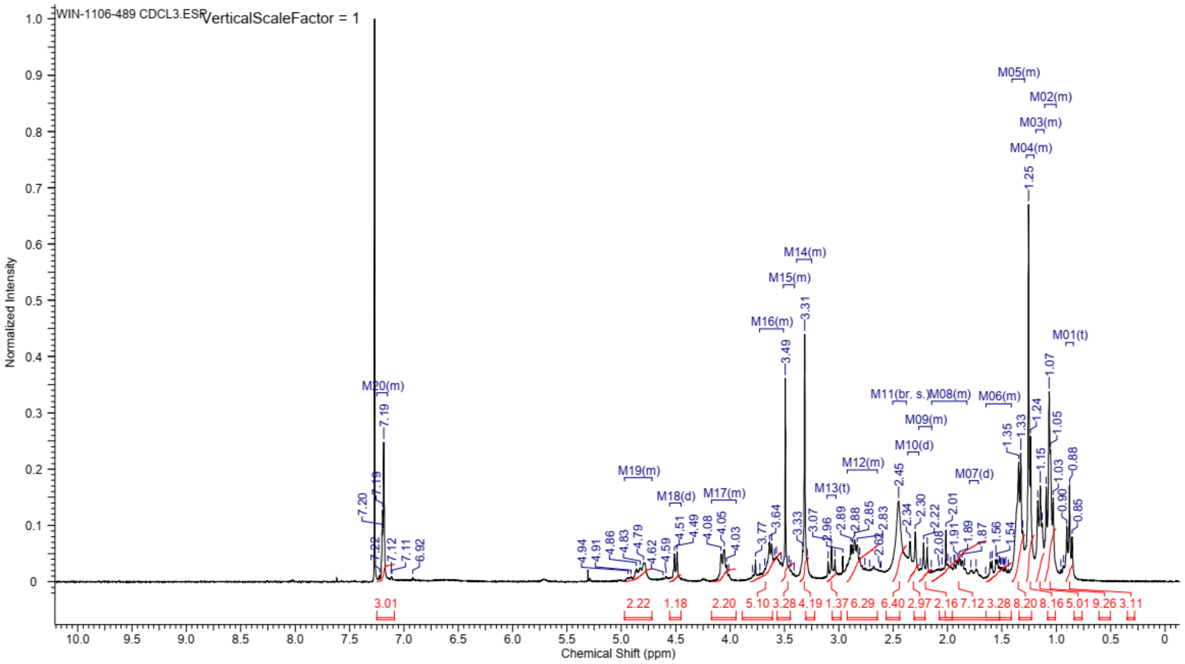
WIN-1106-489 CDCL3.ESP
VerticalScaleFactor = 1
Normalized Intensity
Chemical Shift (ppm)
M01(t)
M02(m)
M03(m)
M04(m)
M05(m)
M06(m)
M07(d)
M08(m)
M09(m)
M10(d)
M11(br. s.)
M12(m)
M13(t)
M14(m)
M15(m)
M16(m)
M17(m)
M18(d)
M19(m)
M20(m)
3.01
2.22
1.18
2.20
5.10
3.28
4.19
1.37
6.29
6.40
2.97
2.16
7.12
3.28
8.20
8.16
5.01
9.26
3.11

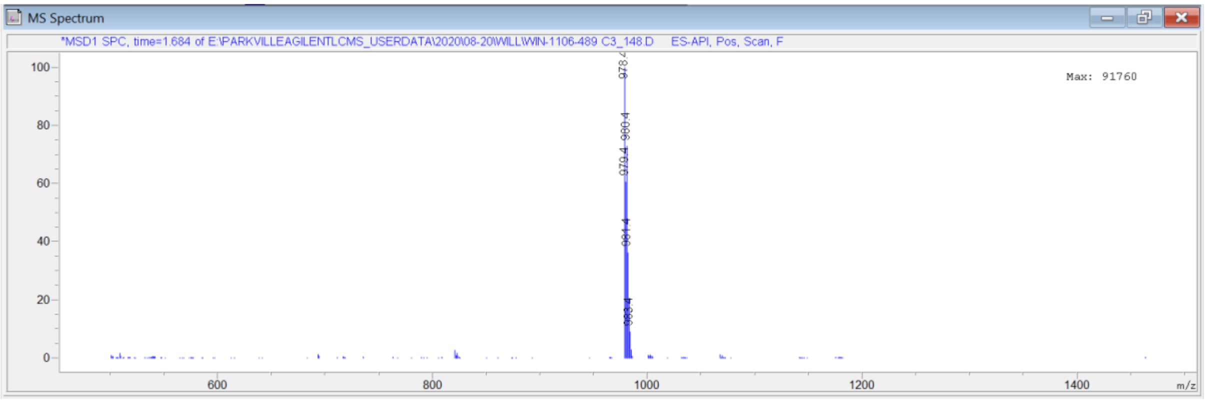
MS Spectrum
*MSD1 SPC, time=1.684 of E:\PARKVILLEAGILENTLCMS_USERDATA\2020\08-20\WILL\WIN-1106-489 C3_148.D ES-API, Pos, Scan, F
Max: 91760
978.4
980.4
979.4
981.4
983.4
m/z

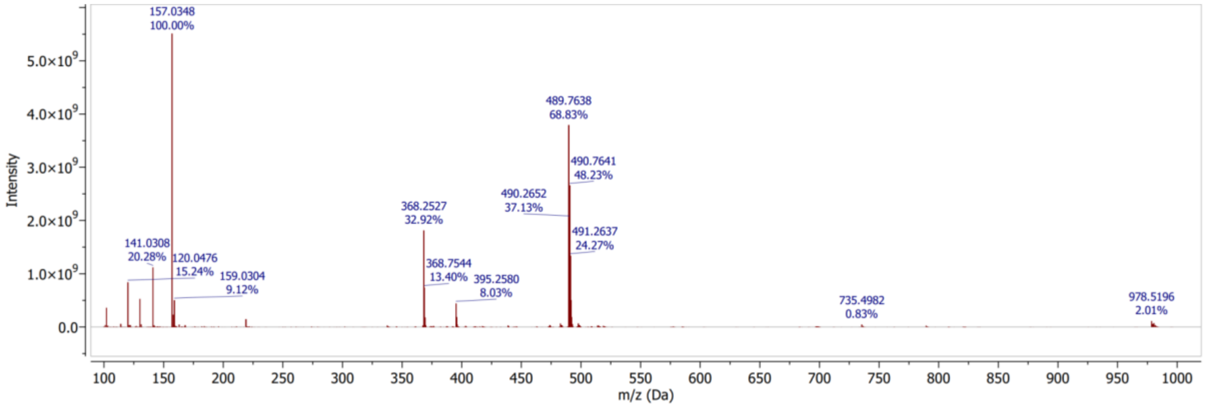
157.0348
100.00%
489.7638
68.83%
490.7641
48.23%
490.2652
37.13%
368.2527
32.92%
491.2637
24.27%
141.0308
20.28%
120.0476
15.24%
159.0304
9.12%
368.7544
13.40%
395.2580
8.03%
735.4982
0.83%
978.5196
2.01%
Intensity
m/z (Da)
