# Supplementary material for: Characterizing the quick-killing mechanism of action of azithromycin analogs against malaria parasites
Source: Antimicrob Agents Chemother. 2025 Jul 25;69(9):e01783-24. doi: 10.1128/aac.01783-24 (PMC12406678; doi:10.1128/aac.01783-24)
Supplement: Supplemental figures — Figures S1 to S9. [file aac.01783-24-s0002.pdf]

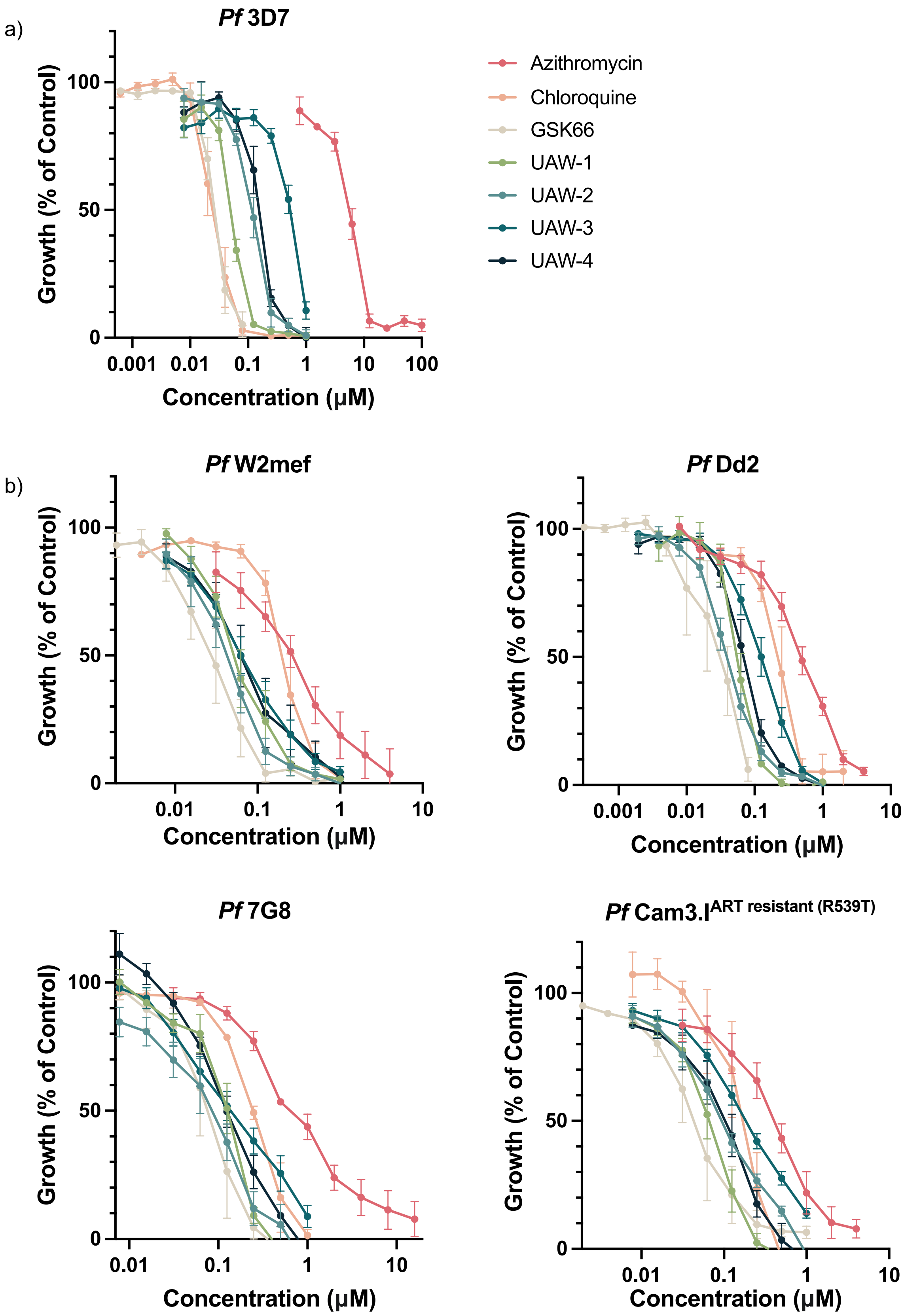
a)
Pf 3D7
Growth (% of Control)
Concentration (µM)
0.001 0.01 0.1 1 10 100
0 50 100
Azithromycin
Chloroquine
GSK66
UAW-1
UAW-2
UAW-3
UAW-4
b)
Pf W2mef
Pf Dd2
Pf 7G8
Pf Cam3.I ART resistant (R539T)
0.01 0.1 1 10
0.001 0.01 0.1 1 10

Figure S1. Growth inhibitory activity of chloroquine, azithromycin and azithromycin analogues against drug sensitive and resistant *P. falciparum* parasites.

Ring-stage 3D7 (a) and multi-drug resistant (b) parasites were drug treated for 72 hrs before growth measurement by DNA staining. All values of growth were normalised to that of parasites grown without drug. Dose response curves were generated in GraphPad Prism, with concentrations along the x-axis adjusted to a $\log_{10}$ scale. Error bars represent the SEM for three or more independent experiments each conducted with technical replicates.

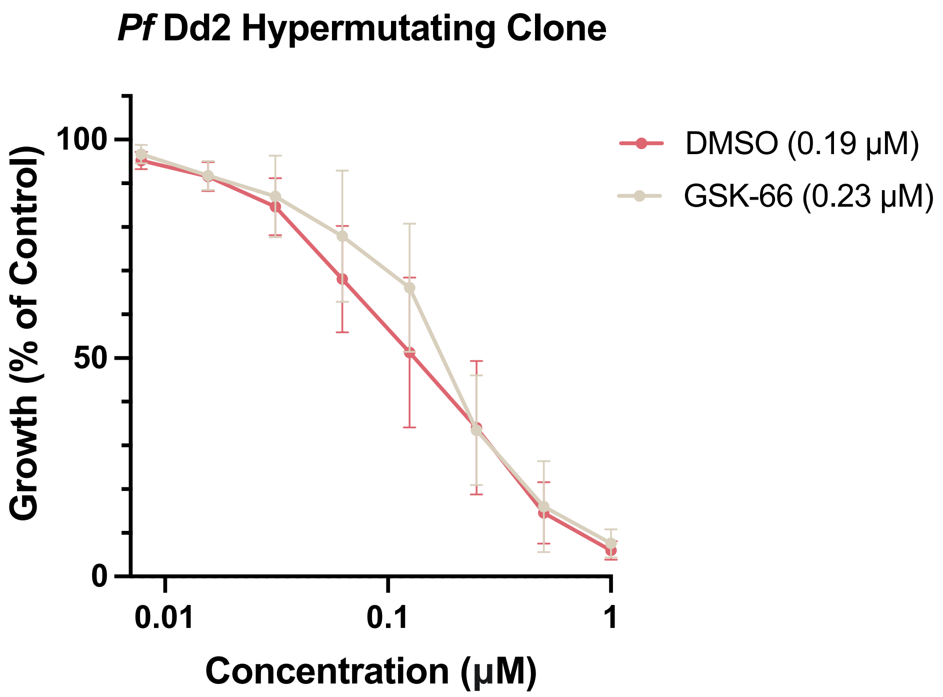

Figure S2. GSK-66 resistance selection in *Pf* Dd2 hypermutating line.

Drug inhibition assays with parasites selected for resistance to increasing drug exposure to GSK-66, or 1:1000 DMSO as a control. IC_{50} values are indicated in the brackets, showing only a 1.5-fold increase in resistance selected parasites. Error bars represent the SEM for three or more independent experiments each conducted with technical replicates.

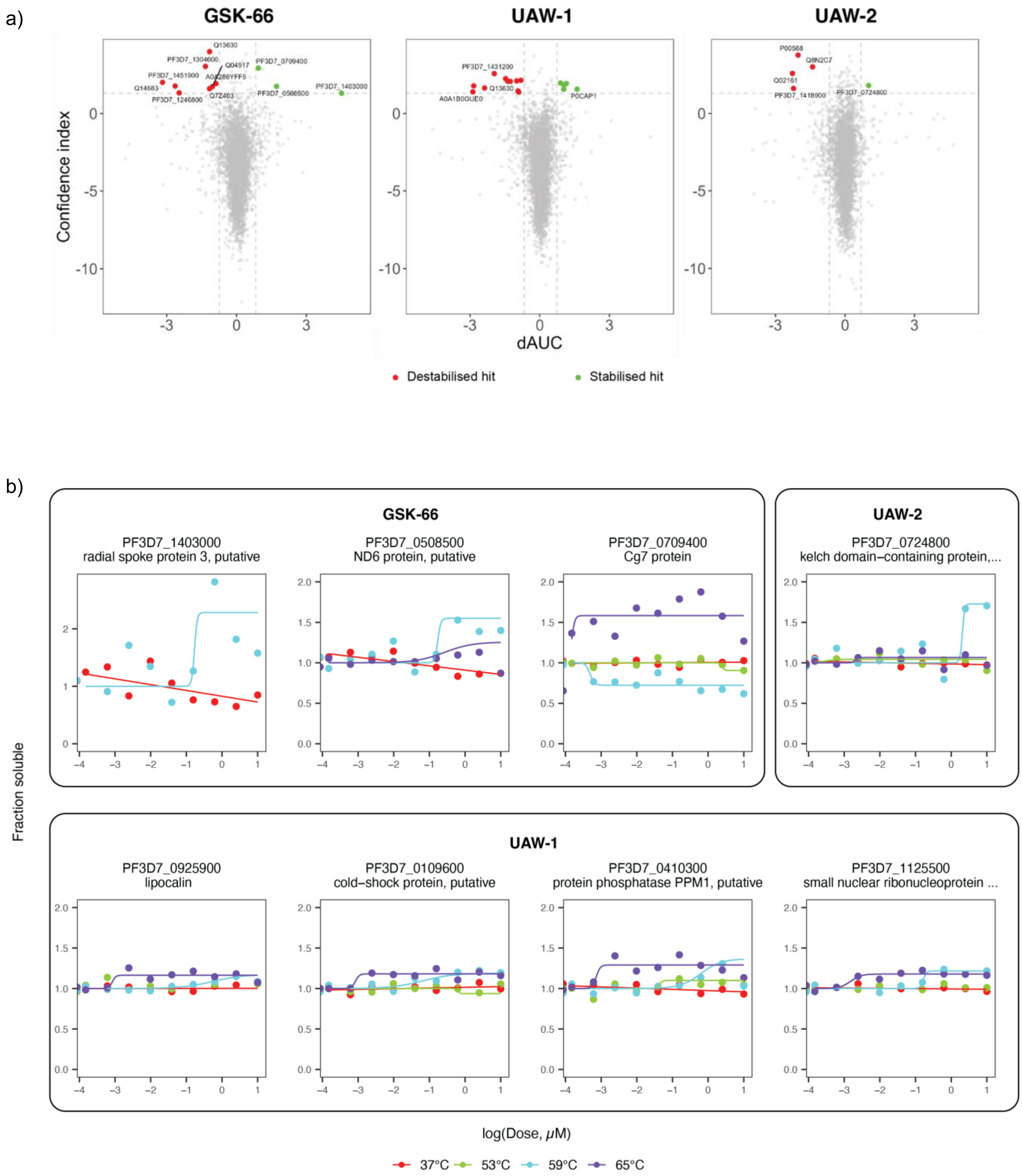
a)
GSK-66
UAW-1
UAW-2
Confidence index
dAUC
Q13630
PF3D7_1304600
Q04917
PF3D7_0709400
PF3D7_1451900
A0A286YFF5
Q14683
PF3D7_0508500
PF3D7_1403000
PF3D7_1246800
Q7Z403
PF3D7_1431200
Q13630
A0A1B0GUE0
P0CAP1
P00568
Q8N2C7
Q02161
PF3D7_1418900
PF3D7_0724800
Destabilised hit
Stabilised hit
b)
GSK-66
UAW-2
PF3D7_1403000
radial spoke protein 3, putative
PF3D7_0508500
ND6 protein, putative
PF3D7_0709400
Cg7 protein
PF3D7_0724800
kelch domain−containing protein,...
Fraction soluble
UAW-1
PF3D7_0925900
lipocalin
PF3D7_0109600
cold−shock protein, putative
PF3D7_0410300
protein phosphatase PPM1, putative
PF3D7_1125500
small nuclear ribonucleoprotein ...
log(Dose, μM)
37°C
53°C
59°C
65°C

Figure S3. Proteins that exhibited a significant thermal shift with drug treatment in lysate CETSA experiments.

(a) Volcano plots showing significantly stabilised and destabilised human and parasite proteins following CETSA with lysate samples of *Pf* 3D7 trophozoites. Dotted lines represent the cut-off criteria with proteins considered as hits when their confidence index was above *-log(0.05)* and the median absolute deviation of ΔAUC was higher than 3. (b) Isothermal dose-response (ITDR) curves for significantly stabilised parasite proteins. While significantly thermal stabilised, these proteins, however, displayed mixed destabilising and stabilising curves at different temperatures, or a low magnitude of response (<1.3). All data representative of one independent experiment.

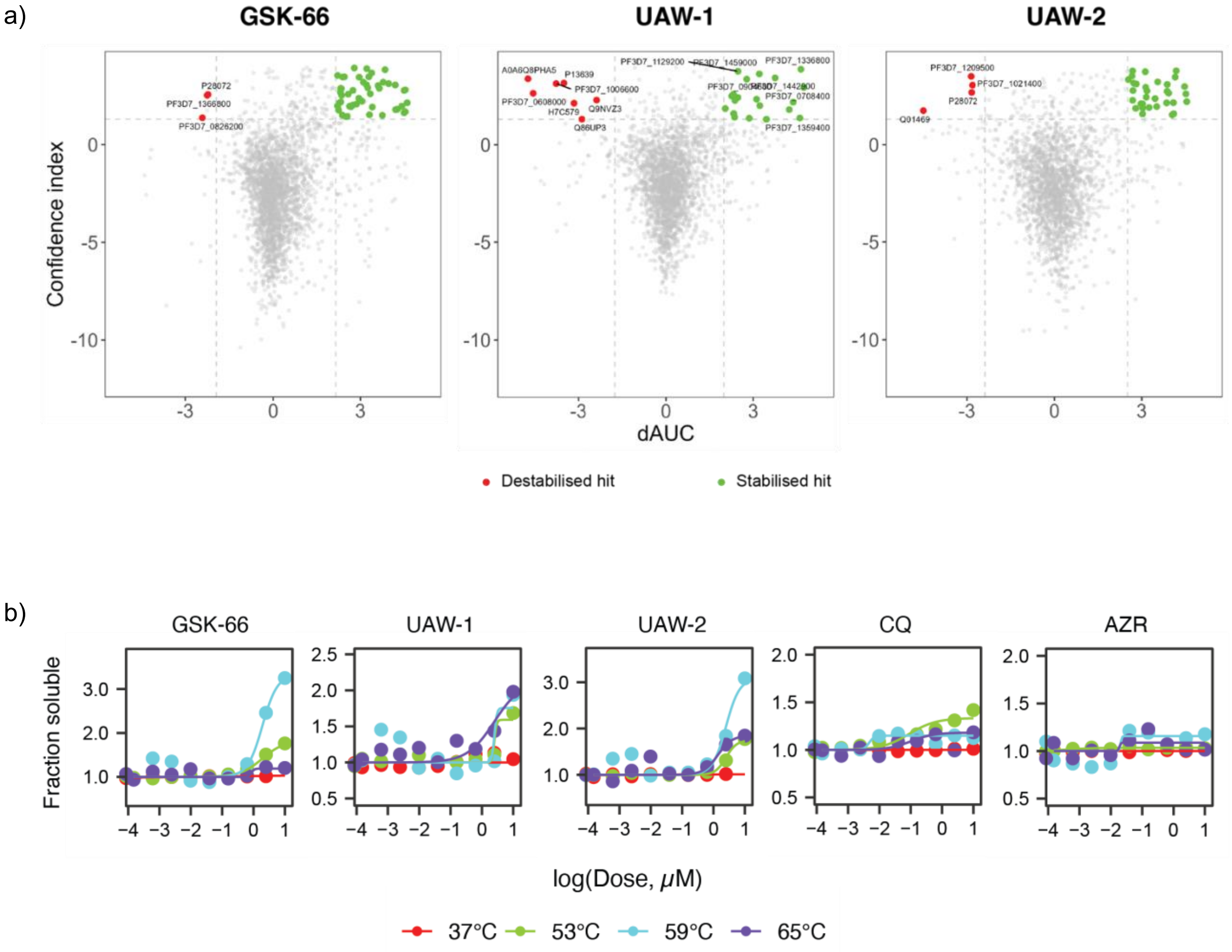

Figure S4. Proteins that exhibited a thermal shift with drug treatment in intact-cell CETSA experiments.

a) Volcano plots showing significantly stabilised and destabilised human and parasite proteins following CETSA of drug-treated *Pf* 3D7 trophozoites. Dotted lines represent the cut-off criteria with proteins considered as hits when their confidence index was above *-log(0.05)* and the median absolute deviation of *ΔAUC* was higher than 3. b) ITDR curves for falcilysin, a previously validated CETSA hit for chloroquine. All but azithromycin demonstrated low levels of thermal

stabilisation relative to the untreated control. Data is representative of at least one independent experiment.

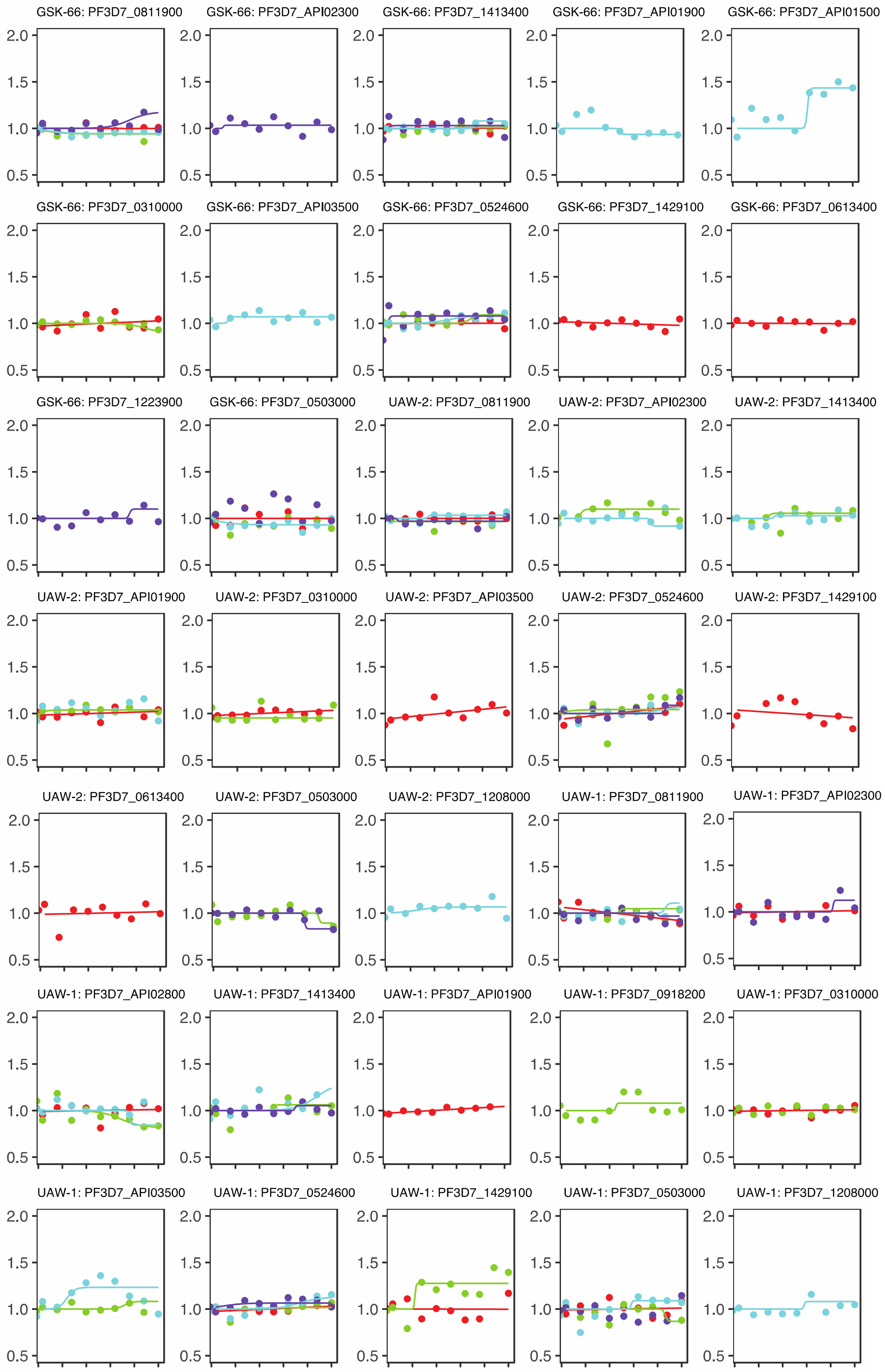
GSK-66: PF3D7_0811900
GSK-66: PF3D7_API02300
GSK-66: PF3D7_1413400
GSK-66: PF3D7_API01900
GSK-66: PF3D7_API01500
GSK-66: PF3D7_0310000
GSK-66: PF3D7_API03500
GSK-66: PF3D7_0524600
GSK-66: PF3D7_1429100
GSK-66: PF3D7_0613400
GSK-66: PF3D7_1223900
GSK-66: PF3D7_0503000
UAW-2: PF3D7_0811900
UAW-2: PF3D7_API02300
UAW-2: PF3D7_1413400
UAW-2: PF3D7_API01900
UAW-2: PF3D7_0310000
UAW-2: PF3D7_API03500
UAW-2: PF3D7_0524600
UAW-2: PF3D7_1429100
UAW-2: PF3D7_0613400
UAW-2: PF3D7_0503000
UAW-2: PF3D7_1208000
UAW-1: PF3D7_0811900
UAW-1: PF3D7_API02300
UAW-1: PF3D7_API02800
UAW-1: PF3D7_1413400
UAW-1: PF3D7_API01900
UAW-1: PF3D7_0918200
UAW-1: PF3D7_0310000
UAW-1: PF3D7_API03500
UAW-1: PF3D7_0524600
UAW-1: PF3D7_1429100
UAW-1: PF3D7_0503000
UAW-1: PF3D7_1208000
2.0
1.5
1.0
0.5

Figure S5. Stabilisation profile of apicoplast proteins detected from lysate CETSA experiments.

Isothermal dose-response (ITDR) curves demonstrate minimal evidence of stabilisation across proteins of the 50s apicoplast ribosome, the known target of azithromycin.

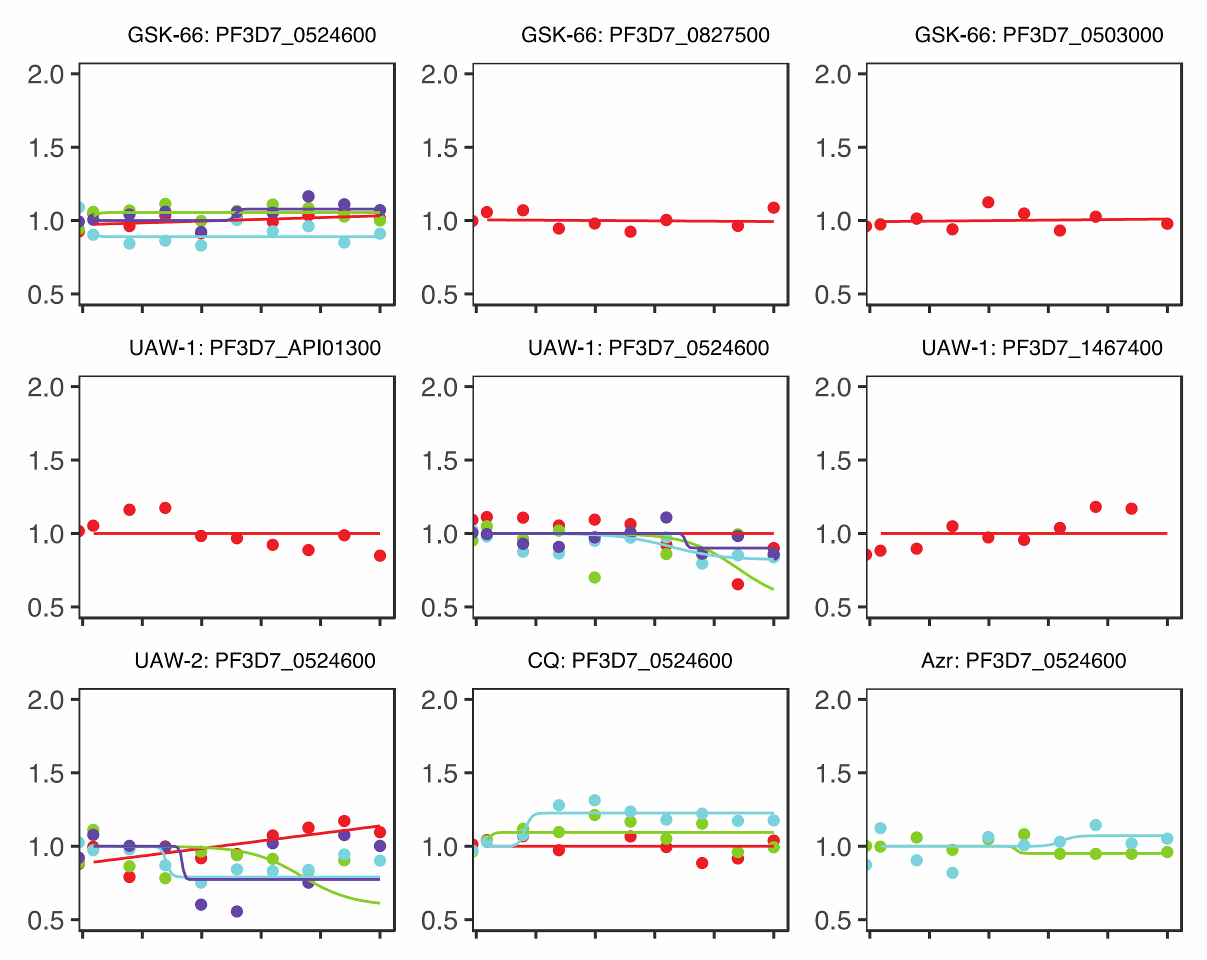

Figure S6. Stabilisation profile of apicoplast proteins detected from intact-cell CETSA experiments.

Isothermal dose-response (ITDR) curves demonstrate minimal evidence of stabilisation across proteins of the 50s apicoplast ribosome, the known target of azithromycin.

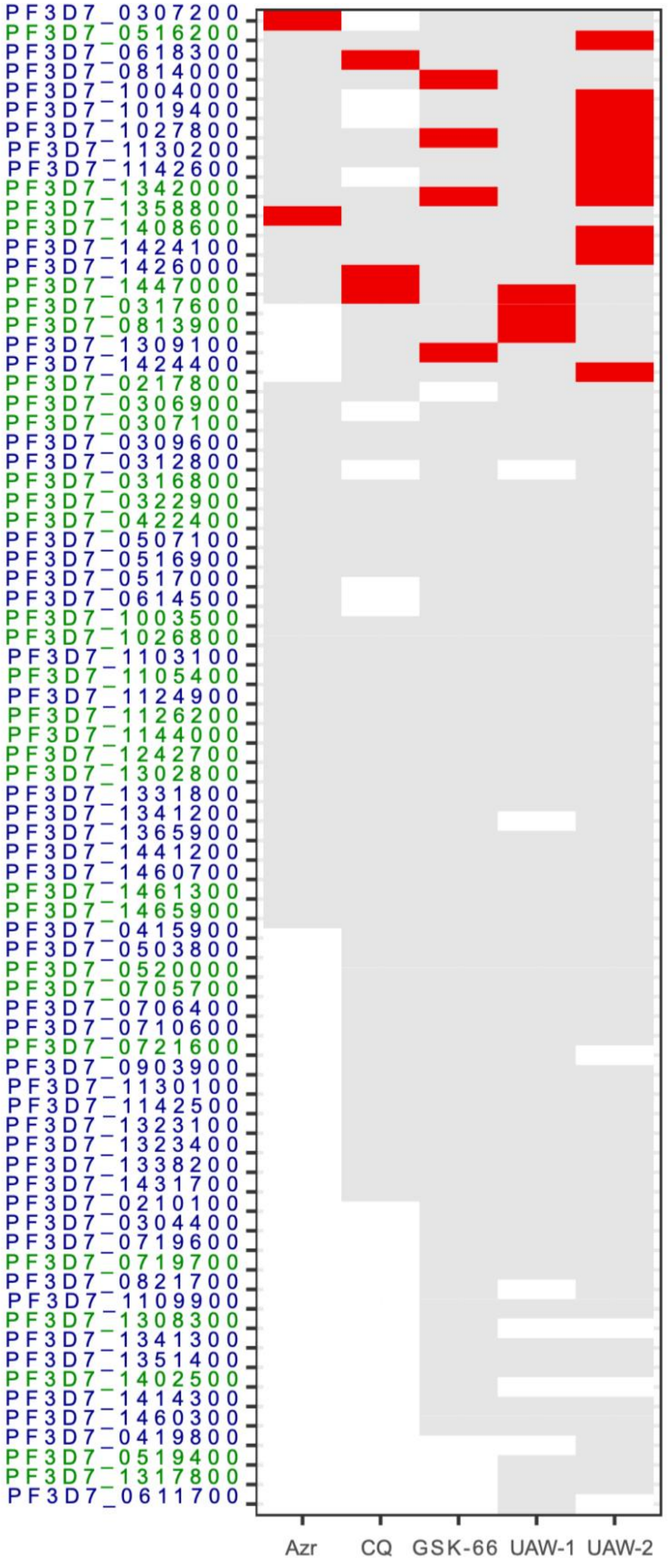
PF3D7_0307200
PF3D7_0516200
PF3D7_0618300
PF3D7_0814000
PF3D7_1004000
PF3D7_1019400
PF3D7_1027800
PF3D7_1130200
PF3D7_1142600
PF3D7_1342000
PF3D7_1358800
PF3D7_1408600
PF3D7_1424100
PF3D7_1426000
PF3D7_1447000
PF3D7_0317600
PF3D7_0813900
PF3D7_1309100
PF3D7_1424400
PF3D7_0217800
PF3D7_0306900
PF3D7_0307100
PF3D7_0309600
PF3D7_0312800
PF3D7_0316800
PF3D7_0322900
PF3D7_0422400
PF3D7_0507100
PF3D7_0516900
PF3D7_0517000
PF3D7_0614500
PF3D7_1003500
PF3D7_1026800
PF3D7_1103100
PF3D7_1105400
PF3D7_1124900
PF3D7_1126200
PF3D7_1144000
PF3D7_1242700
PF3D7_1302800
PF3D7_1331800
PF3D7_1341200
PF3D7_1365900
PF3D7_1441200
PF3D7_1460700
PF3D7_1461300
PF3D7_1465900
PF3D7_0415900
PF3D7_0503800
PF3D7_0520000
PF3D7_0705700
PF3D7_0706400
PF3D7_0710600
PF3D7_0721600
PF3D7_0903900
PF3D7_1130100
PF3D7_1142500
PF3D7_1323100
PF3D7_1323400
PF3D7_1338200
PF3D7_1431700
PF3D7_0210100
PF3D7_0304400
PF3D7_0719600
PF3D7_0719700
PF3D7_0821700
PF3D7_1109900
PF3D7_1308300
PF3D7_1341300
PF3D7_1351400
PF3D7_1402500
PF3D7_1414300
PF3D7_1460300
PF3D7_0419800
PF3D7_0519400
PF3D7_1317800
PF3D7_0611700
Azr
CQ
GSK-66
UAW-1
UAW-2

Figure S7. Cytoplasmic ribosome proteins detected for at least one drug in the intact-cell CETSA.

All proteins in blue are components of the 60s ribosomal subunit, whereas those in green constitute the 40s ribosomal subunit. Proteins that were significantly stabilised in the intact-cell CETSA are indicated in red. Proteins in grey exhibited a small thermal shift upon drug treatment but not within the dAUC cut-off of 3 used in this study to be considered a hit.

a)

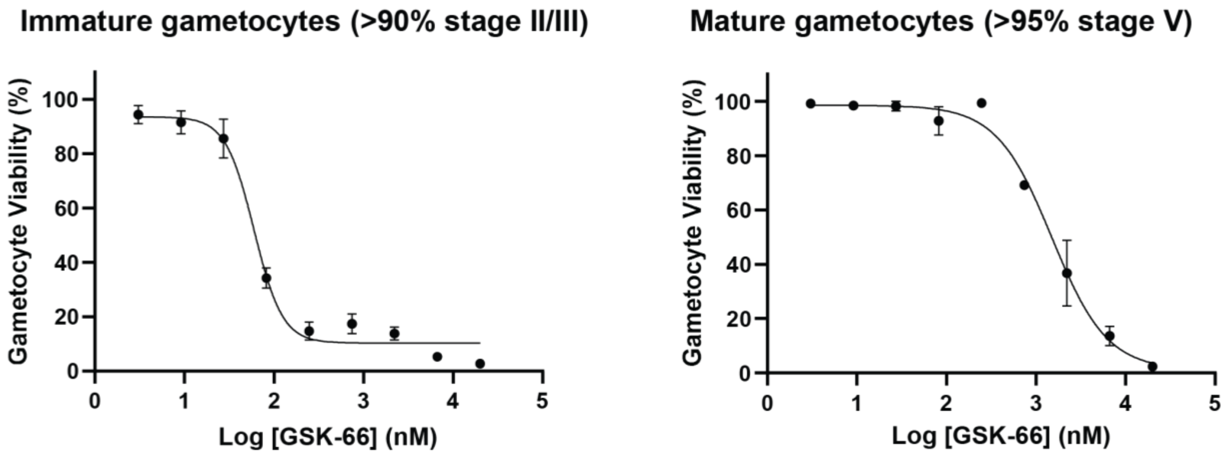

b)

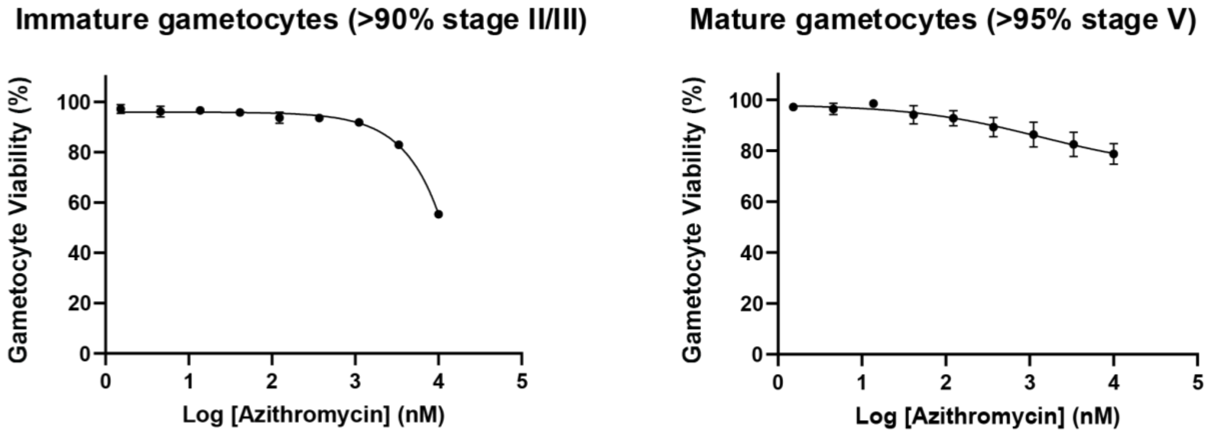

c)

	GSK-66		Azithromycin	
	Immature	Mature	Immature	Mature
**IC_{50} (nM ± SEM)**	62.29 ± 1.89	1478.7 ± 257.8	ND	ND
**Range**	3.05 nM – 20 μM	3.05 nM – 20 μM	1.5 nM – 10 μM	1.5 nM – 10 μM
**IC_{50} n =**	3	3	3	3
**IC_{50} R^2**	0.9671	0.9639	0.9736	0.6331
**Max inhibition (%)**	97.3	97.7	45.2	21.3
**Hill slope**	-2.727	-1.406	-1.014	-0.5542
**IC_{50} z-factor**	0.90	0.82	0.92	0.84
**Methylene blue (% 5 μM)**	94.8	90.3	98.6	68.5
**MMV048 (% 5 μM)**	67.9	65.2	82.9	86.0

Figure S8. Dose response curves of GSK-66 and azithromycin against immature and mature stage gametocytes.

Immature and mature stage gametocytes were treated with GSK-66 (a) or azithromycin (b) for 42 hrs before measurement of luciferase activity. (c) Summary of experimental set-up for IC_{50} determination and quality control parameters to demonstrate inter-assay reliability across independent experiments for GSK-66 and azithromycin. ND = IC_{50} not determined.

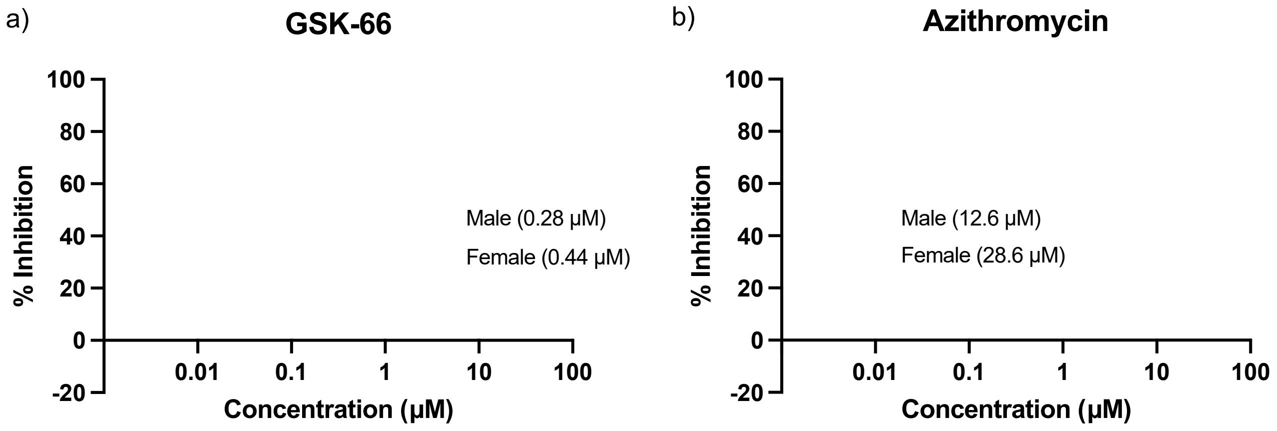

Figure S9. Dose response curves of GSK-66 and azithromycin in Dual Gamete Formation assays (DGFA).

Mature stage V gametocytes were incubated with GSK-66 (a) and azithromycin (b) for 48 hrs before induction of gametogenesis. IC_{50} values are indicated in the brackets, with error bars representing the SEM for four independent experiments.
